# Crystal structures of 3,4,5-tri­phenyl­toluene and 3,4,5-tri­phenyl­benzyl bromide

**DOI:** 10.1107/S2056989025006462

**Published:** 2025-07-31

**Authors:** Pierre Seidel, Eric Meier, Monika Mazik

**Affiliations:** a Institut für Organische Chemie, Technische Universität Bergakademie Freiberg, Leipziger Str. 29, D-09596 Freiberg/Sachsen, Germany; Texas A & M University, USA

**Keywords:** crystal structure, polymorphism, C—H⋯π contacts, van der Waals forces

## Abstract

This article describes the crystal structures of 3,4,5-tri­phenyl­toluene, C_25_H_20_ (**1**), and 3,4,5-tri­phenyl­benzyl bromide, C_25_H_19_Br (**2**), which represent two inter­mediates of a multistep synthesis of a phenyl­acetic acid derivative. Compound **1** crystallizes from methanol in two polymorphic forms, with the space groups *P*2/*n* (**1a**) and *P*2_1_/c (**1b**). In both cases, van der Waals forces significantly contribute to the cohesion of the crystal structure and the two polymorphs are characterized by similar modes of mol­ecular inter­connection. Compound **2** crystallizes from *n*-hexane in the space group *P*1, showing a similar pattern of noncovalent inter­actions to **1a** and **1b**. In all reported structures, the aromatic framework of the mol­ecules adopts a paddlewheel-like conformation.

## Abstract 2

The crystal structures of 3,4,5-tri­phenyl­toluene and 3,4,5-tri­phenyl­benzyl bromide are described, the former of which crystallizes in two polymorphic forms.

## 1. Chemical context

3,4,5-Tri­phenyl­toluene (**1**) and 3,4,5-tri­phenyl­benzyl bromide (**2**) are inter­mediates of a multistep synthesis of 2-(3,4,5-tri­phenyl­phen­yl)acetic acid, which we have recently described (Mazik & Seidel, 2024 ▸; Seidel *et al.*, 2024 ▸). Phenyl­acetic acid and its derivatives are versatile organic compounds, with a variety of valuable properties, including inter­esting biological activities (Cook, 2019 ▸; Jiao *et al.*, 2022 ▸; Perez *et al.*, 2023 ▸). For example, anti-cancer effects can be attributed to 3,4-di­hydroxy­phenyl­acetic acid (Gao *et al.*, 2006 ▸), which is a metab­olite of the neurotransmitter dopamine and other compounds such as rutin (Olthof *et al.*, 2003 ▸), a flavonoid with a diverse pharmacological spectrum (Agrawal *et al.*, 2021 ▸; Mazik, 2022 ▸). Furthermore, it should be mentioned that phenyl­acetic acid is a building block of many well-known medicines, including ibuprofen, diclofenac and flurbiprofen. In addition, phenyl­acetic acid and its derivatives are starting materials for the synthesis of a large number of pharmaceuticals (Vardanyan & Hruby, 2006 ▸). Examples include bendazole, camylofin, triafungin, phenacenide, lorcainide, phenindione, cyclo­pentolate and penicillin.
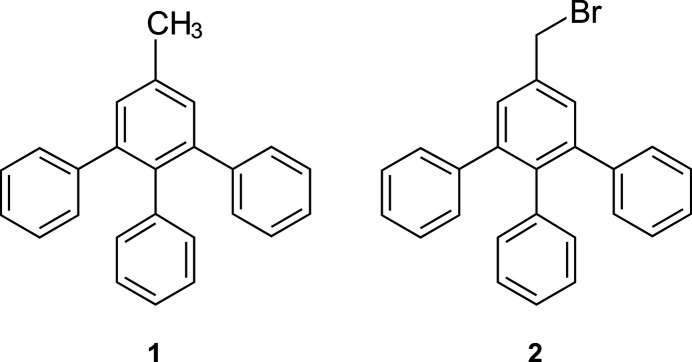


The aforementioned 2-(3,4,5-tri­phenyl­phen­yl)acetic acid and its amide 2-(3,4,5-tri­phenyl­phen­yl)acetamide were synthesized by us as part of studies to develop new anti­carcinogenic substances. In this paper we describe the crystal structures of compounds **1** and **2**. Inter­estingly, two polymorphic forms were found in the case of 3,4,5-tri­phenyl­toluene (**1**).

## 2. Structural commentary

**Figure 1:**
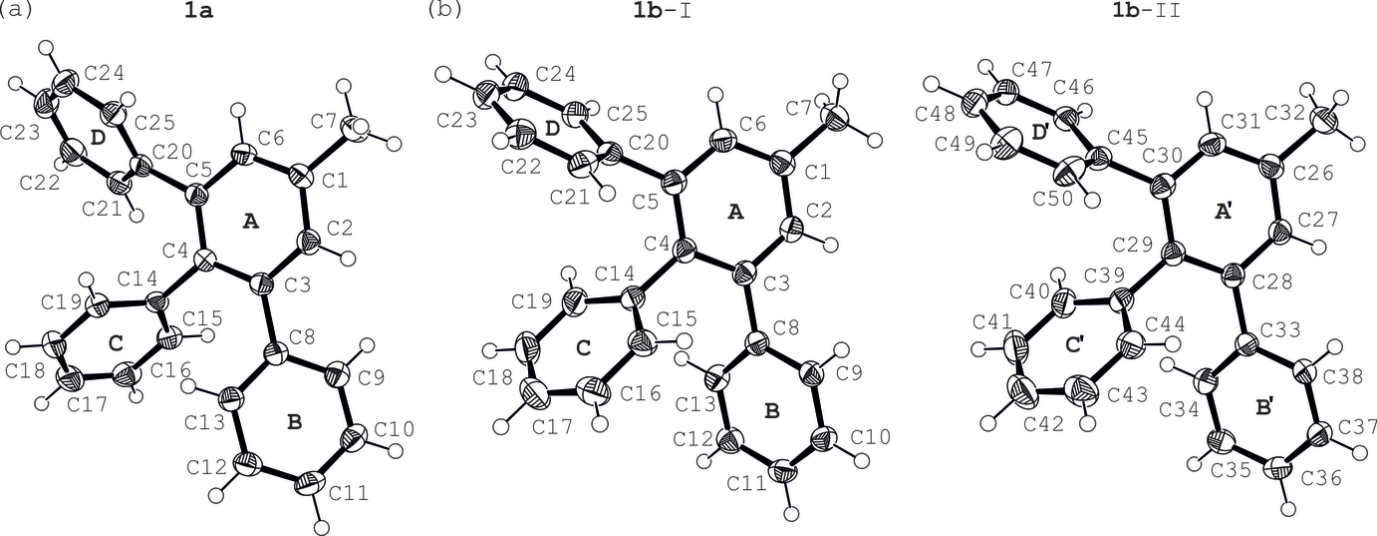
Perspective view of the independent mol­ecules in structures (*a*) **1a** and (*b*) **1b** including atom labelling and ring specification. Displacement ellipsoids are shown at the 50% probability level.

3,4,5-Tri­phenyl­toluene (**1**) crystallizes in two different forms, denoted as **1a** and **1b**. Recrystallization of the compound from methanol yielded colorless blocks of the monoclinic space group *P*2/*n* with one mol­ecule in the asymmetric unit of the cell (**1a**, see Fig. 1 ▸*a*). Leaving the mother liquor to cool further lead to the crystallization of the second polymorphic form (**1b**) in the space group *P*2_1_/c with two independent mol­ecules in the asymmetric unit of the cell (mol­ecules I and II, see Fig. 1 ▸*b*).

Despite these symmetry-related differences, the conformations of the mol­ecules in both crystal structures are similar. In the case of polymorph **1a**, the three phenyl rings (**B**–**D**) are inclined at angles of 40.0 (1), 65.0 (1) and 47.6 (1)° with respect to the plane of the central arene ring (**A**). In the crystal of polymorph **1b**, the analogous angles amount to 57.2 (1)/49.3 (1), 63.9 (1)/63.4 (1) and 60.2 (1)/59.2 (1)° for mol­ecules I and II, respectively, giving rise to a paddlewheel-like arrangement of phenyl groups around the central arene ring.

**Figure 2:**
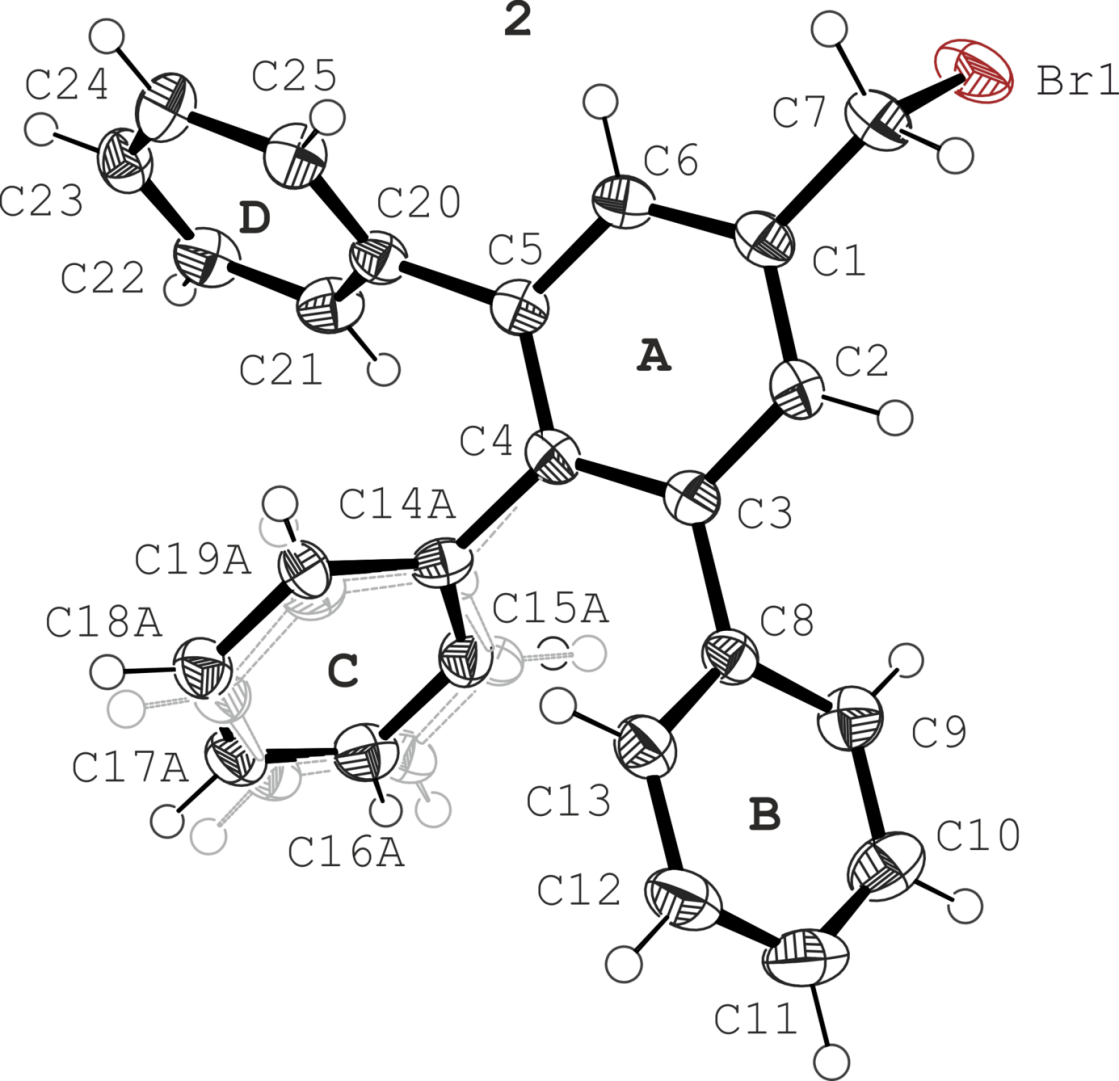
Perspective view of the mol­ecular structure of **2**, with displacement ellipsoids representing the 50% probability level. The ring denoted **C** is disordered over two positions, whereby the minor component is displayed in gray and without labeling.

Crystals of 3,4,5-tri­phenyl­benzyl bromide (**2**) exhibit the space group *P*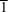 and contain one mol­ecule in the asymmetric unit of the cell (see Fig. 2 ▸). In this structure, the phenyl ring labelled **C** is disordered over two positions with an approximate 50:50 occupancy. Both disordered positions are characterized by similar tilt angles relative to the central arene ring, being 61.6 (3) and 60.7 (3)°. The inclination angles of rings **B** and **D** relative to the central ring (**A**) are 58.2 (1) and 55.2 (1)°, respectively, so that the mol­ecular conformation once again resembles a paddlewheel. The torsion angle given by the atomic sequence C2—C1—C7—Br1 is 95.2 (2)°.

## 3. Supra­molecular features

**Figure 3:**
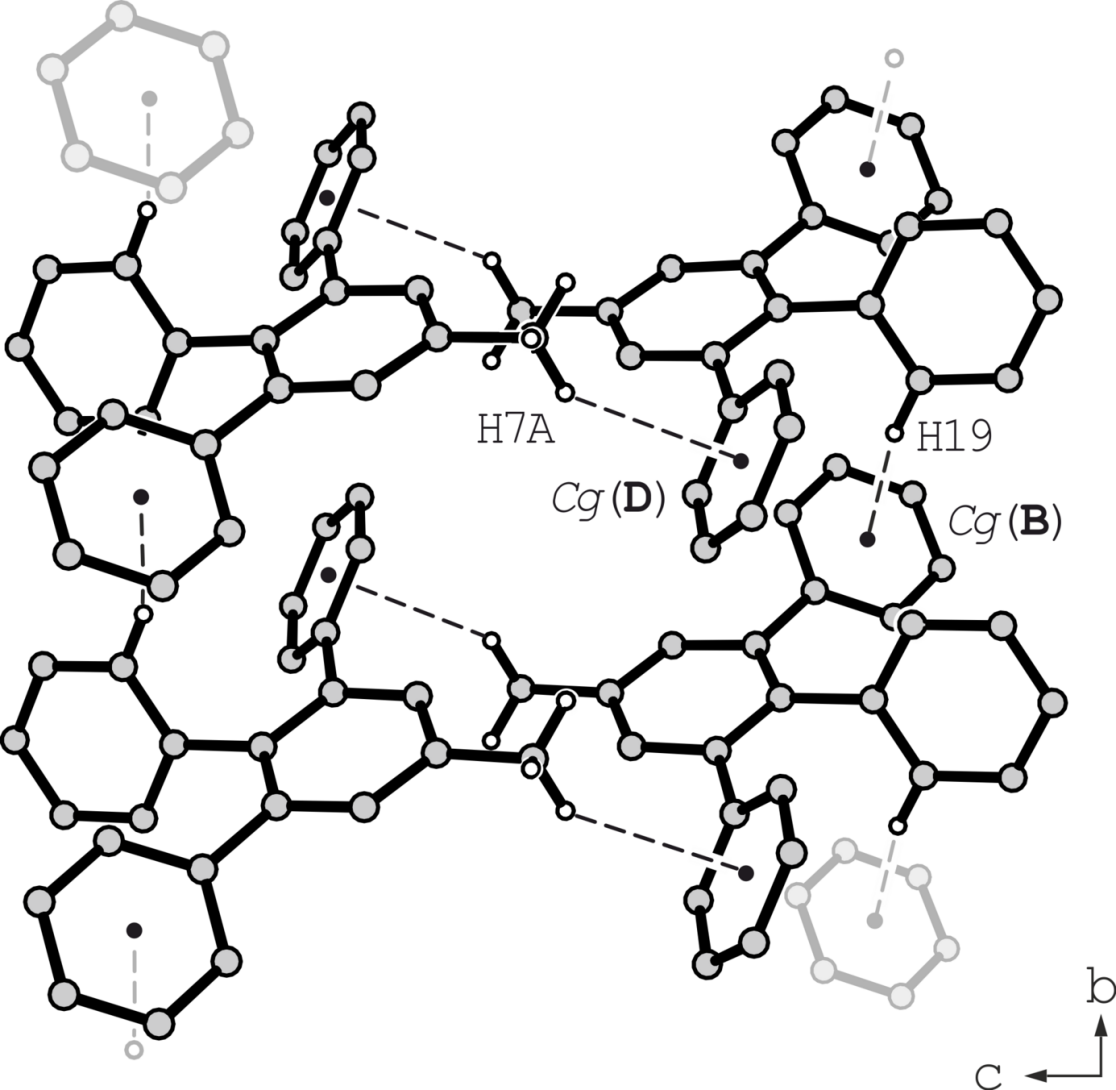
Motif in the crystal structure of **1a** showing the mode of noncovalent inter­molecular bonding. Hydrogen atoms excluded from noncovalent bonding are omitted for clarity.

The two polymorphs of compound **1** are characterized by similar modes of mol­ecular inter­connection, since short directional inter­actions are limited to a few C—H⋯π bonds (see Table 1 ▸). In **1a** this kind of inter­action {*d*[H7*A*⋯*Cg*(**D**)] = 2.87 Å, C—H⋯*Cg* = 134°; *d*[H19⋯*Cg*(**B**)] = 2.97 Å, C—H⋯*Cg* = 145°} generates mono-periodic supra­molecular networks extending parallel to the crystallographic *b*-axis, as shown in Fig. 3 ▸. Since no directional inter­molecular inter­actions between these 1D aggregates are observed, van der Waals forces contribute significantly to the cohesion of the crystal structure.

**Figure 4:**
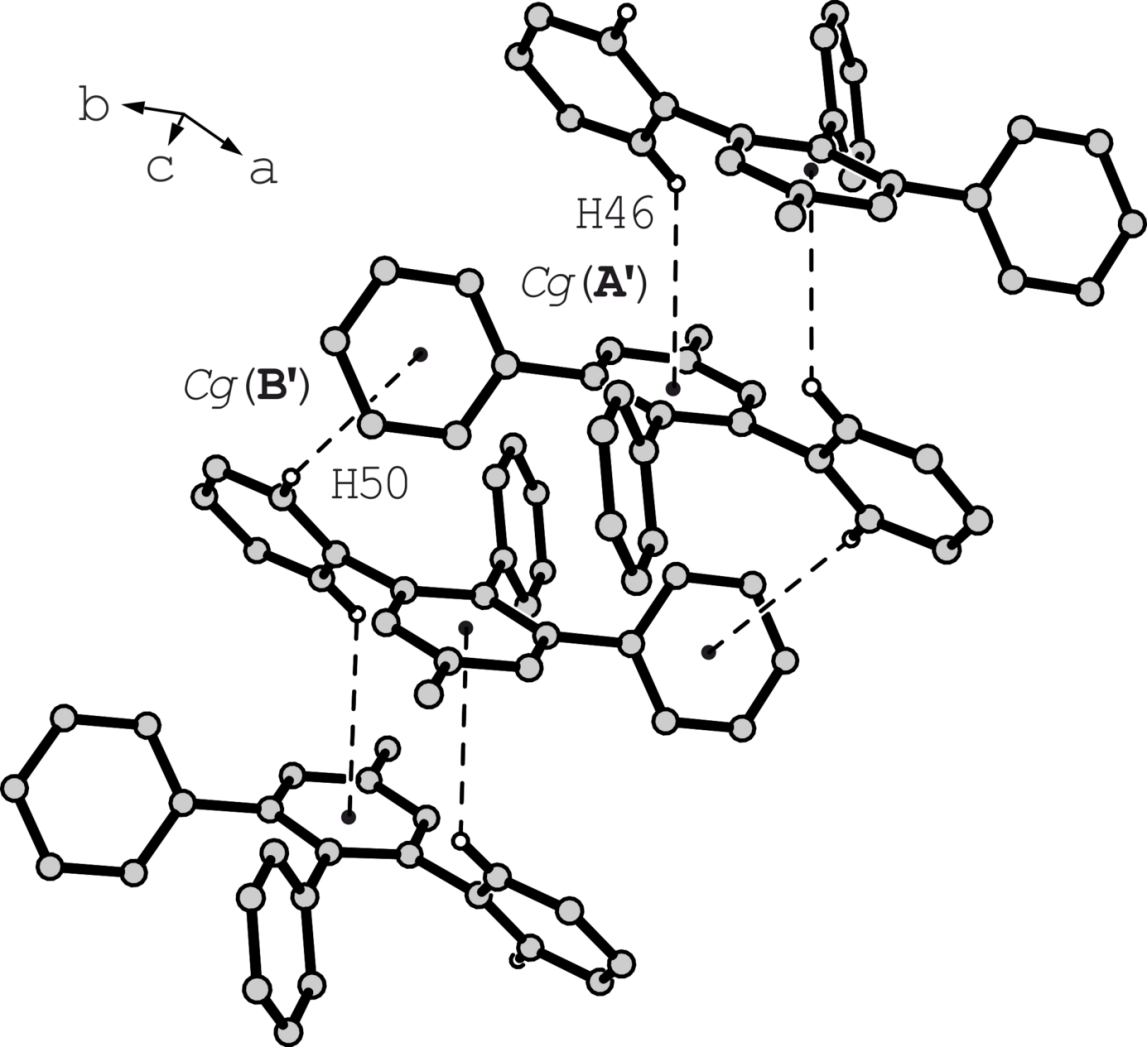
Supra­molecular chains in the crystal structure **1b**. Hydrogen atoms excluded from noncovalent inter­molecular bonding are omitted for clarity.

**Figure 5:**
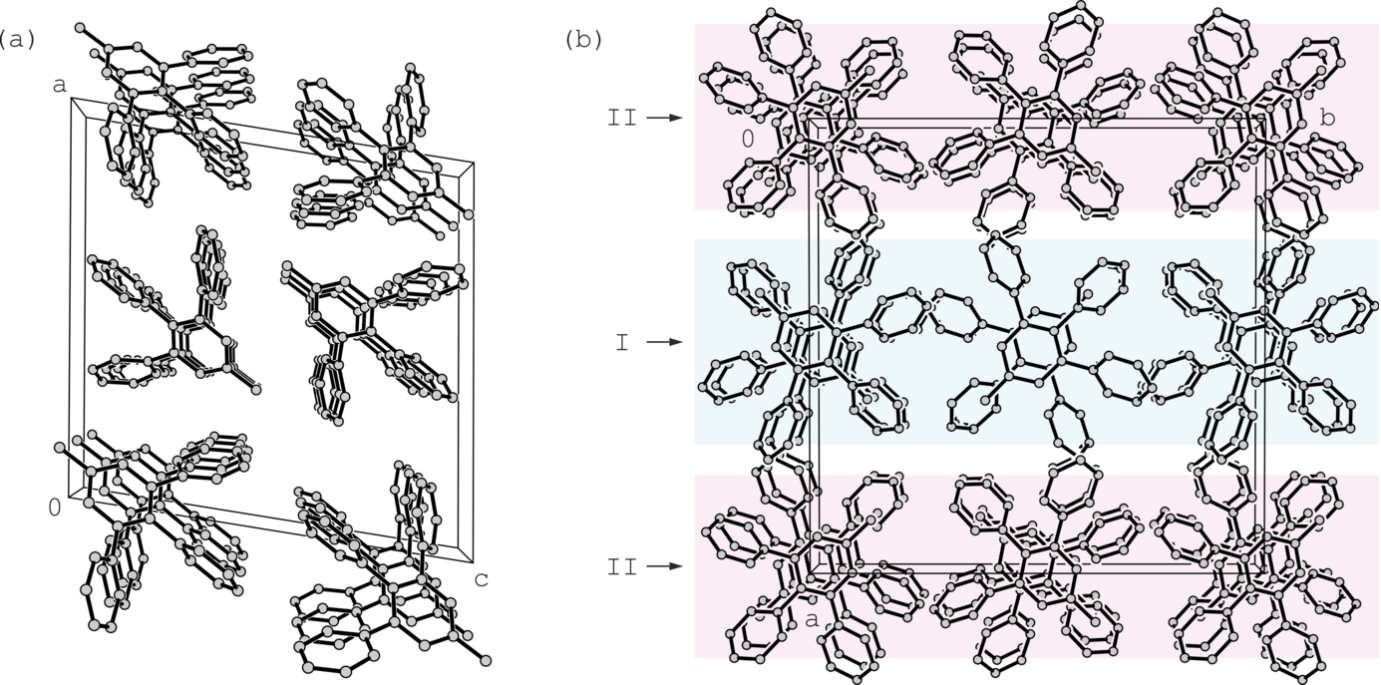
Excerpt of the packing in the crystal structure of (*a*) **1a** and (*b*) **1b** viewed along the crystallographic *b*- and *c*-axis directions, respectively. In the latter case, the structure domains formed by crystallographically non-equivalent mol­ecules I and II are highlighted by different colors. All hydrogen atoms are omitted for clarity.

Similarly, in the crystal structure of the second polymorph of compound **1** (polymorph **1b**), the two crystallographically non-equivalent mol­ecules form linear chain-like aggregates in which the mol­ecules are linked by weak C—H⋯π contacts, as shown in Fig. 4 ▸ for mol­ecule II {*d*[H46⋯*Cg*(**A′**)] = 2.98 Å, C—H⋯*Cg* = 176°; *d*[H50⋯*Cg*(**B′**)] = 2.91 Å, C—H⋯*Cg* = 134°}. The aggregates formed by mol­ecules I and II are structurally similar, each running along the *c*-axis direction. Since no further directional inter­actions are observed in the crystal structure of this polymorph, van der Waals forces are also likely to contribute significantly to its cohesion. Packing differences between the two polymorphs are illustrated in Fig. 5 ▸.

**Figure 6:**
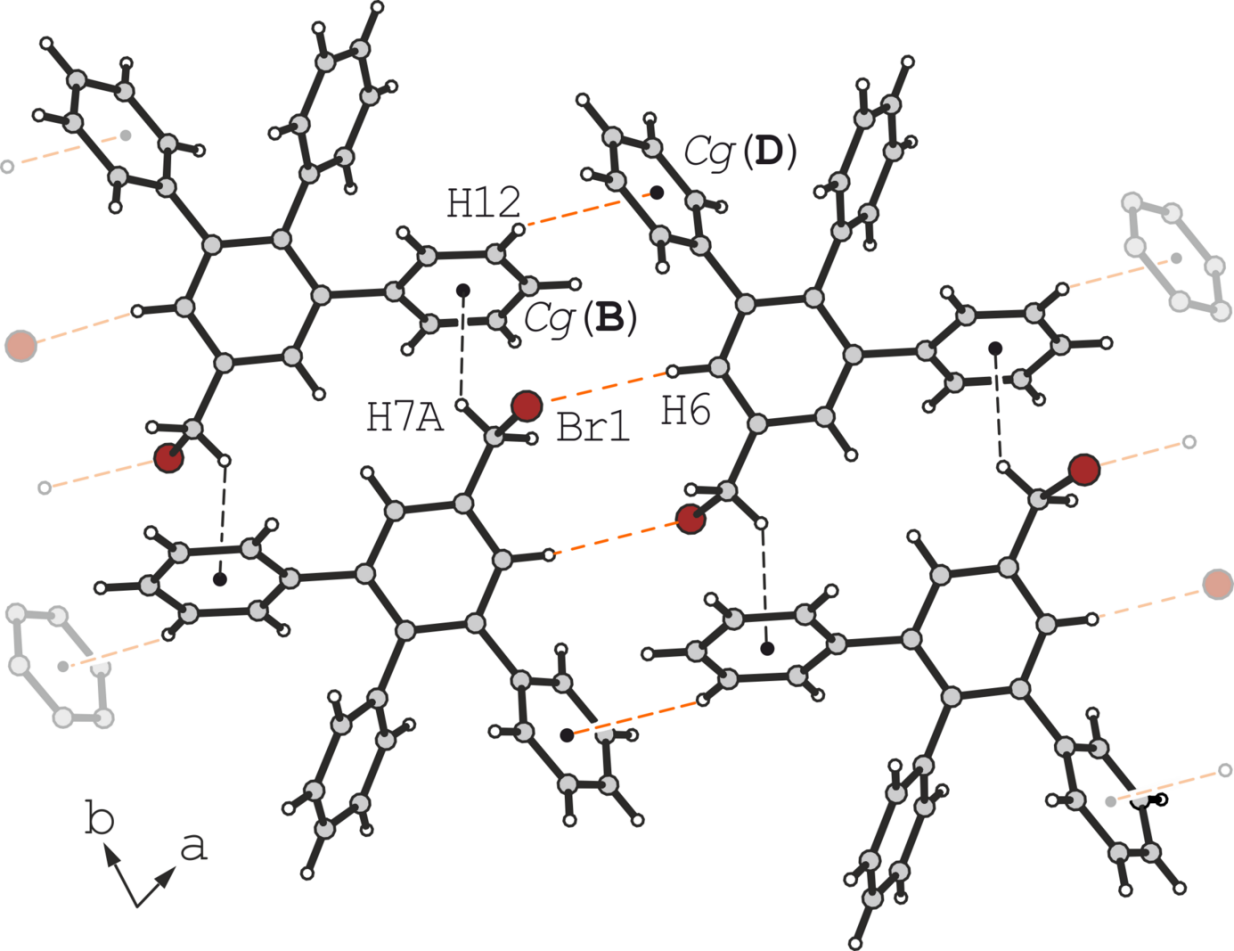
Mode of hydrogen bonding in the crystal structure of **2**. Only the major disorder component of ring **C** is shown. Orange contacts slightly exceed the sum of the van der Waals radii.

**Figure 7:**
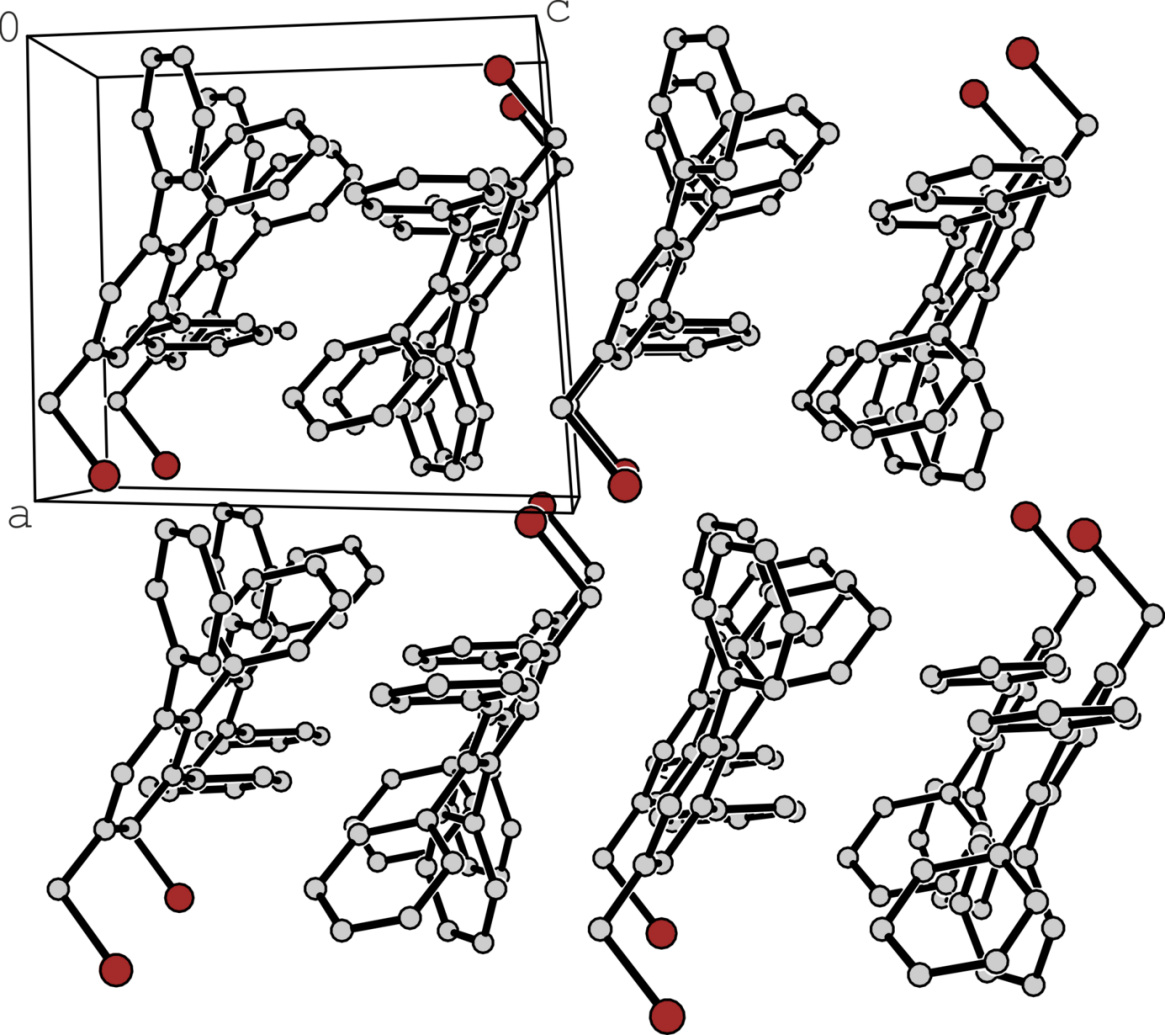
Packing diagram of **2** viewed along the crystallographic *b*-axis direction. Only the major component of the disordered arene ring **C** is shown. All hydrogen atoms are omitted for clarity.

The presence of the additional Br atom in the crystal structure of **2** has little effect on the mode of inter­molecular association. As shown in Fig. 6 ▸ and Table 1 ▸, the crystal structure contains a single short contact, H7*A*⋯*Cg*(**B**) (*d* = 2.81 Å, C—H⋯*Cg* = 138°), grouping mol­ecules into pairs. Other linkage patterns are characterized by distances larger than the van der Waals criterion [*e.g.* H12⋯*Cg*(**D**)]. The same applies to the Br atom, for which the closest neighbour (H6) is located at a distance of 3.11 Å. Although the geometry is almost linear (C—H⋯Br = 170°), the contact distance is slightly above the sum of the van der Waals radii according to Bondi (3.05 Å; Bondi, 1964 ▸). The corresponding packing diagram is shown in Fig. 7 ▸.

## 4. Database survey

A search conducted in the Cambridge Structural Database (CSD, Version 5.46, updated November 2024; Groom *et al.*, 2016 ▸) for methyl and halogenomethyl benzene derivatives with one to five phenyl substituents on the benzene ring only revealed the crystal structure of 1-methyl-2,3,4,5,6-penta­phenyl­benzene (PUNVAW; Gagnon *et al.*, 2010 ▸). Crystallographic studies have been published for methyl and halogenomethyl benzenes with fewer than five phenyl substituents, but the structures most similar to **1** or **2** are not published in the CSD database (for example, 5′-methyl-1,1′:3′,1′′-terphenyl; Hofer & Peebles, 1951 ▸).

The structure PUNVAW is a solvate structure with two mol­ecules of 1-methyl-2,3,4,5,6-penta­phenyl­benzene in two different conformations and half a benzene mol­ecule in the asymmetric unit. The phenyl substituents on the central benzene ring of all host mol­ecules exhibit a paddlewheel arrangement, which is typical for such systems and also occurs in both **1** and **2**. The conformations differ in the arrangement of the phenyl substituents relative to the plane of the central benzene ring. While the basic sense of rotation remains the same, the substituents are arranged more steeply in one conformation than in the other. The crystal structure is mainly characterized by C—H⋯π inter­actions involving the phenyl substituents in positions 1, 2 and 3, or 1 and 3, respectively, of the central benzene ring.

The mol­ecules of one conformer are additionally linked by a C—H⋯π inter­action between the central benzene ring and a phenyl substituent of a second mol­ecule. The remaining substituents participate only in intra­molecular C-H⋯π inter­actions. This also applies to the methyl group, which exerts no discernible influence on the packing. Only a weak van der Waals inter­action with the enclosed solvent is likely.

## 5. Synthesis and crystallization

Compounds **1** and **2** were prepared as previously described (Mazik & Seidel, 2024 ▸). Recrystallization of **1** from methanol yielded polymorph **1a**, while polymorph **1b** slowly crystallized from the respective mother liquor after further cooling. Crystals of **2** were acquired through recrystallization from *n*-hexane.

## 6. Refinement

Crystal data, data collection and structure refinement details are summarized in Table 2 ▸. All non-hydrogen atoms were refined anisotropically, while hydrogen atoms were positioned geometrically and refined isotropically using a riding model [*U*_iso_(H_arene_) = *U*_iso_(H_methyl­ene_) = 1.2 *U*_eq_(C); *U*_iso_(H_meth­yl_) = 1.5 *U*_eq_(C)]. C—H bond distances were set to 0.95 Å (arene), 0.98 Å (meth­yl) and 0.99 Å (methyl­ene), respectively.

## Supplementary Material

Crystal structure: contains datablock(s) 1a, 1b, 2. DOI: 10.1107/S2056989025006462/jy2062sup1.cif

Structure factors: contains datablock(s) 1a. DOI: 10.1107/S2056989025006462/jy20621asup2.hkl

Structure factors: contains datablock(s) 1b. DOI: 10.1107/S2056989025006462/jy20621bsup3.hkl

Structure factors: contains datablock(s) 2. DOI: 10.1107/S2056989025006462/jy20622sup4.hkl

CCDC references: 2473995, 2473994, 2473993

Additional supporting information:  crystallographic information; 3D view; checkCIF report

## Figures and Tables

**Table 1:** Geometric data (A, °) of inter­molecular inter­actions *Cg* denotes the centers of gravity of aromatic rings corresponding to the following atoms: **A′**: C26–C31; **B**: C8–C13; **B′**: C33–C38; **C**: C14–C19; **D**: C20–C25.

C—H⋯Br/*Cg*	C—H	H⋯Br/*Cg*	C⋯Br/*Cg*	C—H⋯Br/*Cg*
**1a**				
C7—H7*A*⋯*Cg*(**D**)^i^	0.98	2.87	3.625 (1)	134
C13—H13⋯*Cg*(**C**)^ii^	0.95	2.97	3.536 (1)	120
C19—H19⋯*Cg*(**B**)^iii^	0.95	2.97	3.790 (1)	145
				
**1b**				
C46—H46⋯*Cg*(**A′**)^iv^	0.95	2.98	3.931 (1)	176
C50—H50⋯*Cg*(**B′**)^v^	0.95	2.91	3.631 (1)	134
				
**2**				
C6—H6⋯Br1^vi^	0.95	3.11^*a*^	4.045 (2)	170
C7—H7*A*⋯*Cg*(**B**)^vii^	0.99	2.81	3.608 (2)	138
C12—H12⋯*Cg*(**D**)^viii^	0.95	3.03^*a*^	3.846 (2)	144

Note: (*a*) distances slightly above commonly employed thresholds (H⋯Br: 3.05 Å, H⋯*Cg*: 3.00 Å; Bondi, 1964 ▸). Symmetry codes: (i) −*x* + 1, −*y* + 1, −*z* + 1; (ii) *x*, *y*, *z* (intra); (iii) *x*, *y* + 1, *z*; (iv) −*x*, −*y* + 1, −*z*; (v) −*x*, −*y* + 1, −*z* + 1; (vi) −*x* + 2, −*y* + 1, −*z*; (vii) −*x* + 1, −*y* + 2, −*z*; (viii) *x* − 1, *y* + 1, *z*.

**Table 2:** Experimental details

	**1a**	**1b**	**2**
Crystal data			
Chemical formula	C_25_H_20_	C_25_H_20_	C_25_H_19_Br
*M*_r_	320.41	320.41	399.31
Crystal system, space group	Monoclinic, *P*2/*n*	Monoclinic, *P*2_1_/*c*	Triclinic, *P* 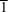
Temperature (K)	163	123	193
*a*, *b*, *c* (Å)	17.0701 (8), 6.0801 (2), 17.4451 (8)	19.9859 (6), 19.9669 (7), 9.2138 (3)	10.0859 (6), 10.2444 (7), 11.3564 (7)
α, β, γ (°)	90, 98.933 (4), 90	90, 94.073 (2), 90	65.309 (5), 76.095 (5), 66.367 (5)
*V* (Å^3^)	1788.63 (13)	3667.5 (2)	972.95 (12)
*Z*	4	8	2
Radiation type	Mo *K*α	Mo *K*α	Mo *K*α
μ (mm^−1^)	0.07	0.07	2.12
Crystal size (mm)	0.22 × 0.12 × 0.06 × 0.09 (radius)	0.2 × 0.19 × 0.18 × 0.16 (radius)	0.50 × 0.40 × 0.25

Data collection			
Diffractometer	Stoe Stadivari	Stoe Stadivari	Stoe *IPDS* 2T
Absorption correction	Multi-scan (*LANA*; Stoe & Cie, 2024*a* ▸)	Multi-scan (*LANA*; Stoe & Cie, 2024*a* ▸)	Integration [*X-SHAPE* (Stoe & Cie, 2021 ▸) and *X-RED32* (Stoe & Cie, 2023 ▸)]
*T*_min_, *T*_max_	0.956, 0.991	0.959, 0.965	0.617, 0.813
No. of measured, independent and observed [*I* > 2σ(*I*)] reflections	36658, 4524, 3319	50810, 7601, 6404	22755, 4702, 4072
*R*_int_	0.038	0.025	0.033
(sin θ/λ)_max_ (Å^−1^)	0.671	0.628	0.660

Refinement			
*R*[*F*^2^ > 2σ(*F*^2^)], *wR*(*F*^2^), *S*	0.043, 0.114, 1.04	0.041, 0.109, 1.05	0.028, 0.069, 1.06
No. of reflections	4524	7601	4702
No. of parameters	227	453	290
No. of restraints	0	0	84
H-atom treatment	H-atom parameters constrained	H-atom parameters constrained	H-atom parameters constrained
Δρ_max_, Δρ_min_ (e Å^−3^)	0.24, −0.23	0.28, −0.25	0.41, −0.38

Computer programs: *X-AREA* (including *Recipe* and *Integrate*) (Stoe & Cie, 2024*b* ▸), *X-RED32* (Stoe & Cie, 2023 ▸), *SHELXT2018/2* (Sheldrick, 2015*a* ▸), *SHELXL2019/3* (Sheldrick, 2015*b* ▸) *OLEX2* (Dolomanov *et al.*, 2009 ▸), *XP* in *SHELXTL* (Sheldrick, 2008 ▸), *ORTEP-3 for Windows* (Farrugia, 2012 ▸) and *publCIF* (Westrip 2010 ▸).

## supplementary crystallographic information

### 5-Methyl-1,2,3-triphenylbenzene (1a) Crystal data

**Table:**

C_25_H_20_	*F*(000) = 680
*M**_r_* = 320.41	*D*_x_ = 1.190 Mg m^−^^3^
Monoclinic, *P*2/*n*	Mo *K*α radiation, λ = 0.71073 Å
*a* = 17.0701 (8) Å	Cell parameters from 27898 reflections
*b* = 6.0801 (2) Å	θ = 1.8–30.8°
*c* = 17.4451 (8) Å	µ = 0.07 mm^−^^1^
β = 98.933 (4)°	*T* = 163 K
*V* = 1788.63 (13) Å^3^	Block, colourless
*Z* = 4	0.22 × 0.12 × 0.06 × 0.09 (radius) mm

### 5-Methyl-1,2,3-triphenylbenzene (1a) Data collection

**Table:**

Stoe Stadivari diffractometer	3319 reflections with *I* > 2σ(*I*)
Detector resolution: 5.81 pixels mm^-1^	*R*_int_ = 0.038
rotation method, ω scans	θ_max_ = 28.5°, θ_min_ = 2.4°
Absorption correction: multi-scan (*LANA*; Stoe & Cie, 2024*a*)	*h* = −22→22
*T*_min_ = 0.956, *T*_max_ = 0.991	*k* = −8→8
36658 measured reflections	*l* = −23→23
4524 independent reflections	

### 5-Methyl-1,2,3-triphenylbenzene (1a) Refinement

**Table:**

Refinement on *F*^2^	Primary atom site location: dual
Least-squares matrix: full	Hydrogen site location: inferred from neighbouring sites
*R*[*F*^2^ > 2σ(*F*^2^)] = 0.043	H-atom parameters constrained
*wR*(*F*^2^) = 0.114	*w* = 1/[σ^2^(*F*_o_^2^) + (0.0547*P*)^2^ + 0.3522*P*] where *P* = (*F*_o_^2^ + 2*F*_c_^2^)/3
*S* = 1.04	(Δ/σ)_max_ < 0.001
4524 reflections	Δρ_max_ = 0.24 e Å^−^^3^
227 parameters	Δρ_min_ = −0.23 e Å^−^^3^
0 restraints	

### 5-Methyl-1,2,3-triphenylbenzene (1a) Special details

**Table:**

Geometry. All esds (except the esd in the dihedral angle between two l.s. planes) are estimated using the full covariance matrix. The cell esds are taken into account individually in the estimation of esds in distances, angles and torsion angles; correlations between esds in cell parameters are only used when they are defined by crystal symmetry. An approximate (isotropic) treatment of cell esds is used for estimating esds involving l.s. planes.

### 5-Methyl-1,2,3-triphenylbenzene (1a) Fractional atomic coordinates and isotropic or equivalent isotropic displacement parameters (Å^2^)

**Table:**

	*x*	*y*	*z*	*U*_iso_*/*U*_eq_	
C1	0.38754 (7)	0.4218 (2)	0.39397 (7)	0.0272 (3)	
C2	0.36343 (7)	0.2941 (2)	0.32872 (7)	0.0273 (3)	
H2	0.318393	0.201679	0.327641	0.033*	
C3	0.40321 (7)	0.29685 (19)	0.26445 (6)	0.0245 (2)	
C4	0.46930 (7)	0.43711 (18)	0.26478 (6)	0.0241 (2)	
C5	0.49296 (7)	0.57198 (19)	0.33007 (7)	0.0251 (2)	
C6	0.45192 (7)	0.5604 (2)	0.39331 (7)	0.0277 (3)	
H6	0.468685	0.650463	0.437302	0.033*	
C7	0.34587 (8)	0.4080 (2)	0.46383 (7)	0.0348 (3)	
H7A	0.358792	0.267807	0.490542	0.052*	
H7B	0.288435	0.417521	0.447182	0.052*	
H7C	0.363252	0.529714	0.499246	0.052*	
C8	0.37152 (7)	0.14970 (19)	0.19821 (7)	0.0252 (2)	
C9	0.34393 (7)	−0.06064 (19)	0.21337 (7)	0.0298 (3)	
H9	0.349558	−0.111702	0.265403	0.036*	
C10	0.30864 (8)	−0.1952 (2)	0.15386 (8)	0.0345 (3)	
H10	0.289642	−0.336307	0.165420	0.041*	
C11	0.30092 (8)	−0.1252 (2)	0.07782 (8)	0.0362 (3)	
H11	0.276543	−0.217542	0.037042	0.043*	
C12	0.32895 (8)	0.0806 (2)	0.06128 (8)	0.0330 (3)	
H12	0.324462	0.128484	0.008945	0.040*	
C13	0.36354 (7)	0.2170 (2)	0.12085 (7)	0.0285 (3)	
H13	0.382074	0.358270	0.108826	0.034*	
C14	0.51397 (7)	0.44349 (19)	0.19752 (7)	0.0255 (3)	
C15	0.55489 (7)	0.2590 (2)	0.17779 (7)	0.0307 (3)	
H15	0.554963	0.128084	0.207509	0.037*	
C16	0.59553 (8)	0.2659 (2)	0.11495 (8)	0.0380 (3)	
H16	0.623412	0.139512	0.101874	0.046*	
C17	0.59577 (9)	0.4551 (3)	0.07120 (8)	0.0426 (4)	
H17	0.623676	0.458773	0.028151	0.051*	
C18	0.55527 (9)	0.6392 (2)	0.09021 (8)	0.0381 (3)	
H18	0.555146	0.769254	0.060020	0.046*	
C19	0.51490 (8)	0.6343 (2)	0.15319 (7)	0.0296 (3)	
H19	0.487649	0.761794	0.166321	0.036*	
C20	0.56168 (7)	0.7268 (2)	0.33745 (7)	0.0270 (3)	
C21	0.63667 (8)	0.6655 (2)	0.32259 (7)	0.0321 (3)	
H21	0.644495	0.522794	0.302971	0.039*	
C22	0.70009 (9)	0.8115 (3)	0.33624 (8)	0.0401 (3)	
H22	0.750985	0.767587	0.326326	0.048*	
C23	0.68919 (9)	1.0207 (3)	0.36424 (8)	0.0435 (4)	
H23	0.732458	1.120536	0.373236	0.052*	
C24	0.61549 (9)	1.0835 (2)	0.37899 (8)	0.0401 (3)	
H24	0.607991	1.226895	0.398235	0.048*	
C25	0.55222 (8)	0.9385 (2)	0.36589 (7)	0.0322 (3)	
H25	0.501671	0.983646	0.376388	0.039*	

### 5-Methyl-1,2,3-triphenylbenzene (1a) Atomic displacement parameters (Å^2^)

**Table:**

	*U*^11^	*U*^22^	*U*^33^	*U*^12^	*U*^13^	*U*^23^
C1	0.0266 (6)	0.0322 (6)	0.0230 (5)	0.0018 (5)	0.0043 (5)	0.0017 (5)
C2	0.0248 (6)	0.0292 (6)	0.0282 (6)	−0.0022 (5)	0.0049 (5)	0.0014 (5)
C3	0.0245 (6)	0.0251 (6)	0.0235 (6)	0.0010 (5)	0.0022 (4)	0.0002 (4)
C4	0.0245 (6)	0.0249 (6)	0.0229 (5)	0.0014 (4)	0.0043 (4)	0.0007 (4)
C5	0.0259 (6)	0.0255 (6)	0.0236 (5)	0.0003 (5)	0.0027 (5)	0.0011 (5)
C6	0.0296 (6)	0.0301 (6)	0.0228 (6)	−0.0006 (5)	0.0024 (5)	−0.0030 (5)
C7	0.0330 (7)	0.0459 (8)	0.0269 (6)	−0.0043 (6)	0.0084 (5)	−0.0025 (6)
C8	0.0209 (6)	0.0270 (6)	0.0281 (6)	0.0005 (5)	0.0051 (5)	−0.0031 (5)
C9	0.0283 (6)	0.0282 (6)	0.0337 (7)	0.0007 (5)	0.0071 (5)	−0.0007 (5)
C10	0.0323 (7)	0.0271 (6)	0.0450 (8)	−0.0030 (5)	0.0094 (6)	−0.0061 (5)
C11	0.0333 (7)	0.0362 (7)	0.0393 (7)	−0.0052 (6)	0.0056 (6)	−0.0153 (6)
C12	0.0322 (7)	0.0399 (7)	0.0271 (6)	−0.0016 (6)	0.0054 (5)	−0.0059 (5)
C13	0.0275 (6)	0.0289 (6)	0.0293 (6)	−0.0015 (5)	0.0051 (5)	−0.0031 (5)
C14	0.0231 (6)	0.0299 (6)	0.0232 (5)	−0.0045 (5)	0.0026 (4)	−0.0043 (5)
C15	0.0287 (6)	0.0319 (6)	0.0315 (6)	−0.0009 (5)	0.0046 (5)	−0.0039 (5)
C16	0.0333 (7)	0.0417 (7)	0.0412 (7)	−0.0008 (6)	0.0126 (6)	−0.0125 (6)
C17	0.0424 (8)	0.0547 (9)	0.0348 (7)	−0.0092 (7)	0.0192 (6)	−0.0075 (7)
C18	0.0435 (8)	0.0409 (7)	0.0315 (7)	−0.0098 (6)	0.0110 (6)	0.0021 (6)
C19	0.0311 (7)	0.0298 (6)	0.0285 (6)	−0.0035 (5)	0.0061 (5)	−0.0014 (5)
C20	0.0303 (6)	0.0300 (6)	0.0196 (5)	−0.0038 (5)	0.0009 (5)	0.0019 (5)
C21	0.0318 (7)	0.0380 (7)	0.0268 (6)	−0.0045 (5)	0.0051 (5)	−0.0020 (5)
C22	0.0310 (7)	0.0559 (9)	0.0328 (7)	−0.0094 (6)	0.0031 (6)	0.0028 (6)
C23	0.0431 (8)	0.0460 (8)	0.0379 (8)	−0.0212 (7)	−0.0047 (6)	0.0045 (6)
C24	0.0503 (9)	0.0302 (7)	0.0357 (7)	−0.0090 (6)	−0.0064 (6)	0.0008 (6)
C25	0.0355 (7)	0.0304 (6)	0.0286 (6)	−0.0005 (5)	−0.0016 (5)	0.0016 (5)

### 5-Methyl-1,2,3-triphenylbenzene (1a) Geometric parameters (Å, º)

**Table:**

C1—C2	1.3865 (17)	C12—C13	1.3878 (17)
C1—C6	1.3864 (17)	C13—H13	0.9500
C1—C7	1.5058 (16)	C14—C15	1.3924 (17)
C2—H2	0.9500	C14—C19	1.3959 (17)
C2—C3	1.3982 (16)	C15—H15	0.9500
C3—C4	1.4135 (16)	C15—C16	1.3861 (18)
C3—C8	1.4948 (16)	C16—H16	0.9500
C4—C5	1.4107 (16)	C16—C17	1.381 (2)
C4—C14	1.4959 (15)	C17—H17	0.9500
C5—C6	1.3975 (16)	C17—C18	1.383 (2)
C5—C20	1.4940 (17)	C18—H18	0.9500
C6—H6	0.9500	C18—C19	1.3849 (17)
C7—H7A	0.9800	C19—H19	0.9500
C7—H7B	0.9800	C20—C21	1.3955 (18)
C7—H7C	0.9800	C20—C25	1.3980 (17)
C8—C9	1.4021 (17)	C21—H21	0.9500
C8—C13	1.3964 (17)	C21—C22	1.3915 (19)
C9—H9	0.9500	C22—H22	0.9500
C9—C10	1.3840 (18)	C22—C23	1.385 (2)
C10—H10	0.9500	C23—H23	0.9500
C10—C11	1.380 (2)	C23—C24	1.377 (2)
C11—H11	0.9500	C24—H24	0.9500
C11—C12	1.3858 (19)	C24—C25	1.3853 (19)
C12—H12	0.9500	C25—H25	0.9500
			
C2—C1—C7	121.07 (11)	C8—C13—H13	119.5
C6—C1—C2	117.91 (11)	C12—C13—C8	120.99 (12)
C6—C1—C7	121.01 (11)	C12—C13—H13	119.5
C1—C2—H2	118.9	C15—C14—C4	120.62 (11)
C1—C2—C3	122.17 (11)	C15—C14—C19	118.91 (11)
C3—C2—H2	118.9	C19—C14—C4	120.47 (10)
C2—C3—C4	119.39 (10)	C14—C15—H15	119.9
C2—C3—C8	116.76 (10)	C16—C15—C14	120.21 (12)
C4—C3—C8	123.85 (10)	C16—C15—H15	119.9
C3—C4—C14	121.08 (10)	C15—C16—H16	119.8
C5—C4—C3	118.83 (10)	C17—C16—C15	120.46 (13)
C5—C4—C14	120.09 (10)	C17—C16—H16	119.8
C4—C5—C20	123.94 (10)	C16—C17—H17	120.1
C6—C5—C4	119.49 (11)	C16—C17—C18	119.82 (12)
C6—C5—C20	116.55 (10)	C18—C17—H17	120.1
C1—C6—C5	122.17 (11)	C17—C18—H18	119.9
C1—C6—H6	118.9	C17—C18—C19	120.13 (13)
C5—C6—H6	118.9	C19—C18—H18	119.9
C1—C7—H7A	109.5	C14—C19—H19	119.8
C1—C7—H7B	109.5	C18—C19—C14	120.46 (12)
C1—C7—H7C	109.5	C18—C19—H19	119.8
H7A—C7—H7B	109.5	C21—C20—C5	123.13 (11)
H7A—C7—H7C	109.5	C21—C20—C25	118.13 (11)
H7B—C7—H7C	109.5	C25—C20—C5	118.58 (11)
C9—C8—C3	119.47 (10)	C20—C21—H21	119.7
C13—C8—C3	122.75 (10)	C22—C21—C20	120.65 (13)
C13—C8—C9	117.67 (11)	C22—C21—H21	119.7
C8—C9—H9	119.4	C21—C22—H22	119.9
C10—C9—C8	121.16 (12)	C23—C22—C21	120.15 (14)
C10—C9—H9	119.4	C23—C22—H22	119.9
C9—C10—H10	119.9	C22—C23—H23	120.1
C11—C10—C9	120.29 (12)	C24—C23—C22	119.82 (13)
C11—C10—H10	119.9	C24—C23—H23	120.1
C10—C11—H11	120.2	C23—C24—H24	119.9
C10—C11—C12	119.61 (12)	C23—C24—C25	120.29 (13)
C12—C11—H11	120.2	C25—C24—H24	119.9
C11—C12—H12	119.9	C20—C25—H25	119.5
C11—C12—C13	120.28 (12)	C24—C25—C20	120.96 (13)
C13—C12—H12	119.9	C24—C25—H25	119.5
			
C1—C2—C3—C4	−1.30 (18)	C6—C5—C20—C25	−45.84 (15)
C1—C2—C3—C8	179.43 (11)	C7—C1—C2—C3	−177.20 (11)
C2—C1—C6—C5	−0.88 (18)	C7—C1—C6—C5	178.26 (12)
C2—C3—C4—C5	−0.42 (17)	C8—C3—C4—C5	178.79 (11)
C2—C3—C4—C14	179.42 (11)	C8—C3—C4—C14	−1.37 (17)
C2—C3—C8—C9	−39.15 (16)	C8—C9—C10—C11	0.91 (19)
C2—C3—C8—C13	136.89 (12)	C9—C8—C13—C12	0.46 (18)
C3—C4—C5—C6	1.43 (17)	C9—C10—C11—C12	0.2 (2)
C3—C4—C5—C20	179.96 (11)	C10—C11—C12—C13	−0.9 (2)
C3—C4—C14—C15	−64.33 (16)	C11—C12—C13—C8	0.60 (19)
C3—C4—C14—C19	115.28 (13)	C13—C8—C9—C10	−1.22 (18)
C3—C8—C9—C10	175.02 (11)	C14—C4—C5—C6	−178.41 (11)
C3—C8—C13—C12	−175.64 (11)	C14—C4—C5—C20	0.12 (17)
C4—C3—C8—C9	141.62 (12)	C14—C15—C16—C17	−0.1 (2)
C4—C3—C8—C13	−42.35 (17)	C15—C14—C19—C18	0.60 (18)
C4—C5—C6—C1	−0.79 (18)	C15—C16—C17—C18	0.1 (2)
C4—C5—C20—C21	−49.16 (17)	C16—C17—C18—C19	0.3 (2)
C4—C5—C20—C25	135.58 (12)	C17—C18—C19—C14	−0.7 (2)
C4—C14—C15—C16	179.41 (11)	C19—C14—C15—C16	−0.21 (18)
C4—C14—C19—C18	−179.02 (12)	C20—C5—C6—C1	−179.43 (11)
C5—C4—C14—C15	115.50 (13)	C20—C21—C22—C23	−0.5 (2)
C5—C4—C14—C19	−64.88 (15)	C21—C20—C25—C24	−0.02 (18)
C5—C20—C21—C22	−174.93 (11)	C21—C22—C23—C24	0.4 (2)
C5—C20—C25—C24	175.48 (11)	C22—C23—C24—C25	−0.1 (2)
C6—C1—C2—C3	1.94 (18)	C23—C24—C25—C20	−0.1 (2)
C6—C5—C20—C21	129.41 (13)	C25—C20—C21—C22	0.35 (18)

### 5-Methyl-1,2,3-triphenylbenzene (1b) Crystal data

**Table:**

C_25_H_20_	*F*(000) = 1360
*M**_r_* = 320.41	*D*_x_ = 1.161 Mg m^−^^3^
Monoclinic, *P*2_1_/*c*	Mo *K*α radiation, λ = 0.71073 Å
*a* = 19.9859 (6) Å	Cell parameters from 25627 reflections
*b* = 19.9669 (7) Å	θ = 2.3–29.4°
*c* = 9.2138 (3) Å	µ = 0.07 mm^−^^1^
β = 94.073 (2)°	*T* = 123 K
*V* = 3667.5 (2) Å^3^	Block, colorless
*Z* = 8	0.2 × 0.19 × 0.18 × 0.16 (radius) mm

### 5-Methyl-1,2,3-triphenylbenzene (1b) Data collection

**Table:**

Stoe Stadivari diffractometer	6404 reflections with *I* > 2σ(*I*)
Detector resolution: 5.81 pixels mm^-1^	*R*_int_ = 0.025
rotation method, ω scans	θ_max_ = 26.5°, θ_min_ = 2.3°
Absorption correction: multi-scan (*LANA*; Stoe & Cie, 2024*a*)	*h* = −25→25
*T*_min_ = 0.959, *T*_max_ = 0.965	*k* = −24→25
50810 measured reflections	*l* = −11→11
7601 independent reflections	

### 5-Methyl-1,2,3-triphenylbenzene (1b) Refinement

**Table:**

Refinement on *F*^2^	Primary atom site location: dual
Least-squares matrix: full	Hydrogen site location: inferred from neighbouring sites
*R*[*F*^2^ > 2σ(*F*^2^)] = 0.041	H-atom parameters constrained
*wR*(*F*^2^) = 0.109	*w* = 1/[σ^2^(*F*_o_^2^) + (0.0491*P*)^2^ + 1.1196*P*] where *P* = (*F*_o_^2^ + 2*F*_c_^2^)/3
*S* = 1.05	(Δ/σ)_max_ = 0.001
7601 reflections	Δρ_max_ = 0.28 e Å^−^^3^
453 parameters	Δρ_min_ = −0.25 e Å^−^^3^
0 restraints	

### 5-Methyl-1,2,3-triphenylbenzene (1b) Special details

**Table:**

Geometry. All esds (except the esd in the dihedral angle between two l.s. planes) are estimated using the full covariance matrix. The cell esds are taken into account individually in the estimation of esds in distances, angles and torsion angles; correlations between esds in cell parameters are only used when they are defined by crystal symmetry. An approximate (isotropic) treatment of cell esds is used for estimating esds involving l.s. planes.

### 5-Methyl-1,2,3-triphenylbenzene (1b) Fractional atomic coordinates and isotropic or equivalent isotropic displacement parameters (Å^2^)

**Table:**

	*x*	*y*	*z*	*U*_iso_*/*U*_eq_	
C1	0.43442 (6)	0.43766 (6)	0.23070 (13)	0.0295 (3)	
C2	0.41940 (6)	0.50432 (6)	0.20010 (13)	0.0283 (3)	
H2	0.374507	0.516210	0.170117	0.034*	
C3	0.46810 (6)	0.55428 (6)	0.21200 (13)	0.0263 (3)	
C4	0.53462 (6)	0.53750 (6)	0.25797 (13)	0.0266 (3)	
C5	0.55064 (6)	0.47015 (6)	0.28724 (13)	0.0273 (3)	
C6	0.50064 (6)	0.42158 (6)	0.27263 (13)	0.0295 (3)	
H6	0.512129	0.376109	0.291872	0.035*	
C7	0.38059 (7)	0.38470 (7)	0.21950 (16)	0.0372 (3)	
H7A	0.400960	0.340567	0.237582	0.056*	
H7B	0.357771	0.385605	0.121797	0.056*	
H7C	0.348019	0.393483	0.291850	0.056*	
C8	0.44871 (6)	0.62421 (6)	0.17107 (13)	0.0263 (3)	
C9	0.48006 (6)	0.65775 (7)	0.06174 (14)	0.0312 (3)	
H9	0.515635	0.636572	0.016214	0.037*	
C10	0.45999 (7)	0.72143 (7)	0.01881 (14)	0.0335 (3)	
H10	0.481312	0.743424	−0.056843	0.040*	
C11	0.40886 (6)	0.75324 (7)	0.08589 (15)	0.0330 (3)	
H11	0.395086	0.797036	0.056631	0.040*	
C12	0.37802 (6)	0.72083 (6)	0.19563 (15)	0.0318 (3)	
H12	0.343396	0.742736	0.242876	0.038*	
C13	0.39735 (6)	0.65642 (6)	0.23729 (14)	0.0285 (3)	
H13	0.375283	0.634241	0.311617	0.034*	
C14	0.58664 (6)	0.59108 (6)	0.27643 (13)	0.0286 (3)	
C15	0.64089 (6)	0.59274 (7)	0.18986 (16)	0.0374 (3)	
H15	0.646070	0.558553	0.119833	0.045*	
C16	0.68751 (7)	0.64434 (9)	0.20571 (18)	0.0475 (4)	
H16	0.724128	0.645562	0.145432	0.057*	
C17	0.68100 (8)	0.69390 (8)	0.30849 (19)	0.0484 (4)	
H17	0.713105	0.728970	0.318815	0.058*	
C18	0.62784 (8)	0.69236 (7)	0.39604 (17)	0.0427 (4)	
H18	0.623449	0.726132	0.467378	0.051*	
C19	0.58082 (7)	0.64135 (7)	0.37956 (14)	0.0335 (3)	
H19	0.544079	0.640690	0.439546	0.040*	
C20	0.62024 (6)	0.44898 (6)	0.33616 (13)	0.0279 (3)	
C21	0.65379 (6)	0.40242 (6)	0.25571 (14)	0.0308 (3)	
H21	0.632503	0.384460	0.168790	0.037*	
C22	0.71813 (7)	0.38208 (7)	0.30172 (15)	0.0347 (3)	
H22	0.740989	0.350880	0.245140	0.042*	
C23	0.74931 (6)	0.40687 (7)	0.42941 (15)	0.0349 (3)	
H23	0.793289	0.392506	0.460844	0.042*	
C24	0.71608 (7)	0.45276 (7)	0.51126 (15)	0.0353 (3)	
H24	0.737019	0.469528	0.599673	0.042*	
C25	0.65231 (7)	0.47412 (7)	0.46398 (14)	0.0326 (3)	
H25	0.630140	0.506333	0.519368	0.039*	
C26	−0.06069 (6)	0.57493 (6)	0.20846 (14)	0.0297 (3)	
C27	0.00534 (6)	0.58734 (6)	0.17877 (13)	0.0279 (3)	
H27	0.016936	0.630202	0.143523	0.033*	
C28	0.05505 (6)	0.53860 (6)	0.19928 (13)	0.0259 (3)	
C29	0.03855 (6)	0.47472 (6)	0.25041 (13)	0.0265 (3)	
C30	−0.02865 (6)	0.46117 (6)	0.27609 (13)	0.0278 (3)	
C31	−0.07677 (6)	0.51114 (7)	0.25494 (13)	0.0300 (3)	
H31	−0.121981	0.501374	0.272803	0.036*	
C32	−0.11277 (7)	0.62934 (7)	0.19349 (17)	0.0391 (3)	
H32A	−0.148790	0.615823	0.121759	0.059*	
H32B	−0.092091	0.670759	0.161238	0.059*	
H32C	−0.131356	0.636896	0.287722	0.059*	
C33	0.12415 (6)	0.55626 (6)	0.16156 (13)	0.0253 (2)	
C34	0.15971 (6)	0.51591 (6)	0.07041 (13)	0.0298 (3)	
H34	0.141303	0.474194	0.038040	0.036*	
C35	0.22134 (6)	0.53576 (7)	0.02654 (14)	0.0329 (3)	
H35	0.244534	0.508133	−0.037168	0.039*	
C36	0.24941 (6)	0.59586 (7)	0.07517 (14)	0.0319 (3)	
H36	0.291627	0.609702	0.044430	0.038*	
C37	0.21566 (6)	0.63542 (7)	0.16847 (15)	0.0326 (3)	
H37	0.235146	0.676238	0.203573	0.039*	
C38	0.15341 (6)	0.61599 (6)	0.21143 (14)	0.0286 (3)	
H38	0.130537	0.643727	0.275495	0.034*	
C39	0.09169 (6)	0.42294 (6)	0.28125 (13)	0.0291 (3)	
C40	0.09113 (7)	0.36267 (7)	0.20527 (16)	0.0366 (3)	
H40	0.056165	0.353661	0.132600	0.044*	
C41	0.14143 (8)	0.31563 (7)	0.23514 (19)	0.0477 (4)	
H41	0.141234	0.275015	0.181403	0.057*	
C42	0.19170 (8)	0.32765 (8)	0.34259 (19)	0.0520 (4)	
H42	0.225510	0.294963	0.363950	0.062*	
C43	0.19285 (8)	0.38723 (8)	0.41908 (17)	0.0455 (4)	
H43	0.227579	0.395624	0.492645	0.055*	
C44	0.14330 (7)	0.43470 (7)	0.38837 (15)	0.0346 (3)	
H44	0.144455	0.475705	0.440779	0.042*	
C45	−0.04948 (6)	0.39414 (6)	0.32844 (13)	0.0285 (3)	
C46	−0.09862 (6)	0.35775 (6)	0.24755 (14)	0.0291 (3)	
H46	−0.118656	0.375635	0.159324	0.035*	
C47	−0.11828 (6)	0.29543 (6)	0.29584 (14)	0.0317 (3)	
H47	−0.151425	0.270637	0.239726	0.038*	
C48	−0.09013 (7)	0.26911 (7)	0.42475 (14)	0.0334 (3)	
H48	−0.103822	0.226435	0.457306	0.040*	
C49	−0.04170 (7)	0.30545 (7)	0.50628 (14)	0.0379 (3)	
H49	−0.022417	0.287812	0.595446	0.046*	
C50	−0.02145 (7)	0.36731 (7)	0.45801 (14)	0.0364 (3)	
H50	0.011997	0.391738	0.514062	0.044*	

### 5-Methyl-1,2,3-triphenylbenzene (1b) Atomic displacement parameters (Å^2^)

**Table:**

	*U*^11^	*U*^22^	*U*^33^	*U*^12^	*U*^13^	*U*^23^
C1	0.0315 (6)	0.0291 (6)	0.0285 (6)	−0.0021 (5)	0.0072 (5)	−0.0024 (5)
C2	0.0252 (6)	0.0304 (6)	0.0294 (6)	0.0016 (5)	0.0036 (5)	−0.0012 (5)
C3	0.0264 (6)	0.0277 (6)	0.0252 (6)	0.0025 (5)	0.0040 (5)	−0.0006 (5)
C4	0.0256 (6)	0.0283 (6)	0.0262 (6)	0.0013 (5)	0.0047 (5)	0.0015 (5)
C5	0.0290 (6)	0.0277 (6)	0.0256 (6)	0.0027 (5)	0.0063 (5)	0.0013 (5)
C6	0.0342 (7)	0.0246 (6)	0.0303 (6)	0.0023 (5)	0.0070 (5)	0.0013 (5)
C7	0.0372 (7)	0.0326 (7)	0.0420 (7)	−0.0060 (6)	0.0039 (6)	0.0005 (6)
C8	0.0241 (6)	0.0266 (6)	0.0277 (6)	−0.0008 (5)	−0.0026 (5)	−0.0023 (5)
C9	0.0290 (6)	0.0343 (7)	0.0302 (6)	0.0035 (5)	0.0016 (5)	0.0006 (5)
C10	0.0348 (7)	0.0331 (7)	0.0322 (7)	−0.0010 (6)	−0.0001 (5)	0.0053 (5)
C11	0.0347 (7)	0.0242 (6)	0.0387 (7)	−0.0005 (5)	−0.0069 (5)	−0.0011 (5)
C12	0.0291 (6)	0.0268 (6)	0.0393 (7)	0.0013 (5)	0.0010 (5)	−0.0066 (5)
C13	0.0258 (6)	0.0275 (6)	0.0322 (6)	−0.0027 (5)	0.0017 (5)	−0.0028 (5)
C14	0.0245 (6)	0.0281 (6)	0.0327 (6)	0.0021 (5)	−0.0012 (5)	0.0088 (5)
C15	0.0290 (7)	0.0410 (8)	0.0425 (7)	0.0041 (6)	0.0048 (6)	0.0125 (6)
C16	0.0270 (7)	0.0555 (10)	0.0602 (10)	−0.0025 (7)	0.0051 (6)	0.0236 (8)
C17	0.0368 (8)	0.0443 (9)	0.0619 (10)	−0.0137 (7)	−0.0119 (7)	0.0203 (8)
C18	0.0458 (8)	0.0339 (8)	0.0465 (8)	−0.0075 (6)	−0.0103 (7)	0.0075 (6)
C19	0.0341 (7)	0.0303 (7)	0.0355 (7)	−0.0016 (5)	−0.0016 (5)	0.0056 (5)
C20	0.0288 (6)	0.0257 (6)	0.0298 (6)	0.0018 (5)	0.0065 (5)	0.0073 (5)
C21	0.0306 (6)	0.0295 (7)	0.0328 (6)	0.0009 (5)	0.0058 (5)	0.0011 (5)
C22	0.0301 (7)	0.0335 (7)	0.0414 (7)	0.0048 (5)	0.0098 (6)	0.0006 (6)
C23	0.0276 (6)	0.0379 (7)	0.0392 (7)	0.0048 (5)	0.0028 (5)	0.0093 (6)
C24	0.0366 (7)	0.0379 (7)	0.0309 (6)	0.0018 (6)	0.0001 (5)	0.0049 (6)
C25	0.0372 (7)	0.0315 (7)	0.0297 (6)	0.0064 (6)	0.0056 (5)	0.0028 (5)
C26	0.0275 (6)	0.0307 (7)	0.0306 (6)	0.0018 (5)	−0.0003 (5)	−0.0078 (5)
C27	0.0287 (6)	0.0249 (6)	0.0297 (6)	−0.0011 (5)	−0.0004 (5)	−0.0030 (5)
C28	0.0270 (6)	0.0247 (6)	0.0258 (6)	−0.0019 (5)	0.0009 (5)	−0.0030 (5)
C29	0.0276 (6)	0.0259 (6)	0.0259 (6)	−0.0020 (5)	0.0022 (5)	−0.0034 (5)
C30	0.0296 (6)	0.0283 (6)	0.0256 (6)	−0.0048 (5)	0.0031 (5)	−0.0048 (5)
C31	0.0247 (6)	0.0349 (7)	0.0307 (6)	−0.0040 (5)	0.0036 (5)	−0.0075 (5)
C32	0.0325 (7)	0.0374 (8)	0.0473 (8)	0.0056 (6)	0.0025 (6)	−0.0085 (6)
C33	0.0247 (6)	0.0247 (6)	0.0263 (6)	0.0006 (5)	−0.0005 (5)	0.0037 (5)
C34	0.0320 (6)	0.0286 (6)	0.0288 (6)	−0.0011 (5)	0.0016 (5)	−0.0004 (5)
C35	0.0303 (7)	0.0388 (7)	0.0298 (6)	0.0029 (5)	0.0040 (5)	−0.0003 (5)
C36	0.0233 (6)	0.0384 (7)	0.0339 (7)	−0.0017 (5)	0.0011 (5)	0.0071 (6)
C37	0.0275 (6)	0.0274 (6)	0.0421 (7)	−0.0023 (5)	−0.0027 (5)	0.0018 (5)
C38	0.0266 (6)	0.0251 (6)	0.0340 (6)	0.0023 (5)	0.0008 (5)	0.0004 (5)
C39	0.0302 (6)	0.0262 (6)	0.0318 (6)	−0.0013 (5)	0.0087 (5)	0.0040 (5)
C40	0.0425 (8)	0.0272 (7)	0.0412 (7)	−0.0018 (6)	0.0110 (6)	0.0008 (6)
C41	0.0591 (10)	0.0272 (7)	0.0591 (10)	0.0066 (7)	0.0207 (8)	0.0022 (7)
C42	0.0512 (9)	0.0442 (9)	0.0614 (10)	0.0185 (7)	0.0112 (8)	0.0158 (8)
C43	0.0399 (8)	0.0502 (9)	0.0463 (8)	0.0090 (7)	0.0031 (6)	0.0129 (7)
C44	0.0333 (7)	0.0353 (7)	0.0355 (7)	0.0013 (6)	0.0043 (5)	0.0050 (6)
C45	0.0273 (6)	0.0300 (7)	0.0290 (6)	−0.0040 (5)	0.0079 (5)	−0.0041 (5)
C46	0.0265 (6)	0.0287 (6)	0.0323 (6)	0.0003 (5)	0.0030 (5)	−0.0038 (5)
C47	0.0293 (6)	0.0279 (7)	0.0381 (7)	−0.0031 (5)	0.0047 (5)	−0.0083 (5)
C48	0.0378 (7)	0.0278 (7)	0.0360 (7)	−0.0046 (5)	0.0119 (6)	−0.0029 (5)
C49	0.0446 (8)	0.0415 (8)	0.0279 (6)	−0.0084 (6)	0.0041 (6)	0.0022 (6)
C50	0.0380 (7)	0.0420 (8)	0.0294 (6)	−0.0136 (6)	0.0035 (5)	−0.0028 (6)

### 5-Methyl-1,2,3-triphenylbenzene (1b) Geometric parameters (Å, º)

**Table:**

C1—C2	1.3891 (18)	C26—C27	1.3887 (17)
C1—C6	1.3898 (18)	C26—C31	1.3888 (19)
C1—C7	1.5068 (18)	C26—C32	1.5041 (18)
C2—H2	0.9500	C27—H27	0.9500
C2—C3	1.3925 (17)	C27—C28	1.3939 (17)
C3—C4	1.4067 (16)	C28—C29	1.4070 (17)
C3—C8	1.4905 (17)	C28—C33	1.4902 (16)
C4—C5	1.4042 (17)	C29—C30	1.4066 (17)
C4—C14	1.4930 (17)	C29—C39	1.4949 (17)
C5—C6	1.3922 (18)	C30—C31	1.3899 (18)
C5—C20	1.4924 (17)	C30—C45	1.4919 (17)
C6—H6	0.9500	C31—H31	0.9500
C7—H7A	0.9800	C32—H32A	0.9800
C7—H7B	0.9800	C32—H32B	0.9800
C7—H7C	0.9800	C32—H32C	0.9800
C8—C9	1.3948 (18)	C33—C34	1.3944 (18)
C8—C13	1.3886 (17)	C33—C38	1.3919 (17)
C9—H9	0.9500	C34—H34	0.9500
C9—C10	1.3827 (18)	C34—C35	1.3814 (18)
C10—H10	0.9500	C35—H35	0.9500
C10—C11	1.3855 (19)	C35—C36	1.3855 (19)
C11—H11	0.9500	C36—H36	0.9500
C11—C12	1.3818 (19)	C36—C37	1.3786 (19)
C12—H12	0.9500	C37—H37	0.9500
C12—C13	1.3892 (18)	C37—C38	1.3874 (18)
C13—H13	0.9500	C38—H38	0.9500
C14—C15	1.3913 (18)	C39—C40	1.3918 (18)
C14—C19	1.3927 (19)	C39—C44	1.3959 (19)
C15—H15	0.9500	C40—H40	0.9500
C15—C16	1.390 (2)	C40—C41	1.389 (2)
C16—H16	0.9500	C41—H41	0.9500
C16—C17	1.382 (3)	C41—C42	1.381 (2)
C17—H17	0.9500	C42—H42	0.9500
C17—C18	1.379 (2)	C42—C43	1.382 (2)
C18—H18	0.9500	C43—H43	0.9500
C18—C19	1.3868 (19)	C43—C44	1.385 (2)
C19—H19	0.9500	C44—H44	0.9500
C20—C21	1.3908 (18)	C45—C46	1.3942 (17)
C20—C25	1.3938 (18)	C45—C50	1.3895 (19)
C21—H21	0.9500	C46—H46	0.9500
C21—C22	1.3858 (18)	C46—C47	1.3876 (18)
C22—H22	0.9500	C47—H47	0.9500
C22—C23	1.383 (2)	C47—C48	1.3816 (19)
C23—H23	0.9500	C48—H48	0.9500
C23—C24	1.386 (2)	C48—C49	1.3871 (19)
C24—H24	0.9500	C49—H49	0.9500
C24—C25	1.3846 (18)	C49—C50	1.3831 (19)
C25—H25	0.9500	C50—H50	0.9500
			
C2—C1—C6	117.75 (12)	C27—C26—C31	117.92 (12)
C2—C1—C7	121.04 (12)	C27—C26—C32	120.94 (12)
C6—C1—C7	121.21 (12)	C31—C26—C32	121.12 (12)
C1—C2—H2	119.0	C26—C27—H27	119.1
C1—C2—C3	122.05 (11)	C26—C27—C28	121.80 (12)
C3—C2—H2	119.0	C28—C27—H27	119.1
C2—C3—C4	119.57 (11)	C27—C28—C29	119.77 (11)
C2—C3—C8	118.90 (11)	C27—C28—C33	117.82 (11)
C4—C3—C8	121.50 (11)	C29—C28—C33	122.38 (11)
C3—C4—C14	119.94 (11)	C28—C29—C39	120.70 (11)
C5—C4—C3	118.92 (11)	C30—C29—C28	118.68 (11)
C5—C4—C14	121.13 (11)	C30—C29—C39	120.59 (11)
C4—C5—C20	121.61 (11)	C29—C30—C45	121.24 (11)
C6—C5—C4	119.76 (11)	C31—C30—C29	119.86 (11)
C6—C5—C20	118.63 (11)	C31—C30—C45	118.89 (11)
C1—C6—C5	121.93 (12)	C26—C31—C30	121.91 (12)
C1—C6—H6	119.0	C26—C31—H31	119.0
C5—C6—H6	119.0	C30—C31—H31	119.0
C1—C7—H7A	109.5	C26—C32—H32A	109.5
C1—C7—H7B	109.5	C26—C32—H32B	109.5
C1—C7—H7C	109.5	C26—C32—H32C	109.5
H7A—C7—H7B	109.5	H32A—C32—H32B	109.5
H7A—C7—H7C	109.5	H32A—C32—H32C	109.5
H7B—C7—H7C	109.5	H32B—C32—H32C	109.5
C9—C8—C3	120.69 (11)	C34—C33—C28	121.62 (11)
C13—C8—C3	120.65 (11)	C38—C33—C28	120.02 (11)
C13—C8—C9	118.62 (12)	C38—C33—C34	118.28 (11)
C8—C9—H9	119.6	C33—C34—H34	119.5
C10—C9—C8	120.80 (12)	C35—C34—C33	120.91 (12)
C10—C9—H9	119.6	C35—C34—H34	119.5
C9—C10—H10	119.9	C34—C35—H35	119.9
C9—C10—C11	120.15 (12)	C34—C35—C36	120.19 (12)
C11—C10—H10	119.9	C36—C35—H35	119.9
C10—C11—H11	120.2	C35—C36—H36	120.2
C12—C11—C10	119.55 (12)	C37—C36—C35	119.55 (12)
C12—C11—H11	120.2	C37—C36—H36	120.2
C11—C12—H12	119.8	C36—C37—H37	119.8
C11—C12—C13	120.40 (12)	C36—C37—C38	120.40 (12)
C13—C12—H12	119.8	C38—C37—H37	119.8
C8—C13—C12	120.47 (12)	C33—C38—H38	119.7
C8—C13—H13	119.8	C37—C38—C33	120.64 (12)
C12—C13—H13	119.8	C37—C38—H38	119.7
C15—C14—C4	121.09 (12)	C40—C39—C29	121.47 (12)
C15—C14—C19	118.70 (12)	C40—C39—C44	118.71 (12)
C19—C14—C4	120.20 (11)	C44—C39—C29	119.82 (11)
C14—C15—H15	120.0	C39—C40—H40	119.8
C16—C15—C14	120.08 (15)	C41—C40—C39	120.34 (14)
C16—C15—H15	120.0	C41—C40—H40	119.8
C15—C16—H16	119.7	C40—C41—H41	119.9
C17—C16—C15	120.51 (14)	C42—C41—C40	120.27 (15)
C17—C16—H16	119.7	C42—C41—H41	119.9
C16—C17—H17	120.0	C41—C42—H42	120.0
C18—C17—C16	119.92 (14)	C41—C42—C43	120.02 (14)
C18—C17—H17	120.0	C43—C42—H42	120.0
C17—C18—H18	120.1	C42—C43—H43	120.0
C17—C18—C19	119.76 (15)	C42—C43—C44	119.93 (15)
C19—C18—H18	120.1	C44—C43—H43	120.0
C14—C19—H19	119.5	C39—C44—H44	119.6
C18—C19—C14	121.02 (13)	C43—C44—C39	120.72 (14)
C18—C19—H19	119.5	C43—C44—H44	119.6
C21—C20—C5	120.17 (11)	C46—C45—C30	119.79 (11)
C21—C20—C25	118.74 (12)	C50—C45—C30	121.26 (11)
C25—C20—C5	121.07 (11)	C50—C45—C46	118.95 (12)
C20—C21—H21	119.8	C45—C46—H46	120.0
C22—C21—C20	120.32 (12)	C47—C46—C45	120.05 (12)
C22—C21—H21	119.8	C47—C46—H46	120.0
C21—C22—H22	119.7	C46—C47—H47	119.7
C23—C22—C21	120.50 (12)	C48—C47—C46	120.63 (12)
C23—C22—H22	119.7	C48—C47—H47	119.7
C22—C23—H23	120.2	C47—C48—H48	120.2
C22—C23—C24	119.67 (12)	C47—C48—C49	119.50 (12)
C24—C23—H23	120.2	C49—C48—H48	120.2
C23—C24—H24	120.0	C48—C49—H49	119.9
C25—C24—C23	119.90 (13)	C50—C49—C48	120.12 (13)
C25—C24—H24	120.0	C50—C49—H49	119.9
C20—C25—H25	119.6	C45—C50—H50	119.6
C24—C25—C20	120.84 (12)	C49—C50—C45	120.75 (12)
C24—C25—H25	119.6	C49—C50—H50	119.6
			
C1—C2—C3—C4	−0.76 (18)	C26—C27—C28—C29	0.46 (18)
C1—C2—C3—C8	177.37 (11)	C26—C27—C28—C33	178.81 (11)
C2—C1—C6—C5	1.43 (18)	C27—C26—C31—C30	1.96 (18)
C2—C3—C4—C5	1.64 (17)	C27—C28—C29—C30	1.62 (17)
C2—C3—C4—C14	−177.70 (11)	C27—C28—C29—C39	−176.50 (11)
C2—C3—C8—C9	−120.99 (13)	C27—C28—C33—C34	−128.22 (13)
C2—C3—C8—C13	56.62 (16)	C27—C28—C33—C38	48.42 (16)
C3—C4—C5—C6	−1.00 (17)	C28—C29—C30—C31	−1.89 (17)
C3—C4—C5—C20	179.91 (11)	C28—C29—C30—C45	179.18 (11)
C3—C4—C14—C15	−115.74 (13)	C28—C29—C39—C40	−117.85 (14)
C3—C4—C14—C19	62.99 (16)	C28—C29—C39—C44	62.14 (16)
C3—C8—C9—C10	176.91 (11)	C28—C33—C34—C35	174.45 (11)
C3—C8—C13—C12	−177.98 (11)	C28—C33—C38—C37	−175.25 (11)
C4—C3—C8—C9	57.10 (16)	C29—C28—C33—C34	50.09 (17)
C4—C3—C8—C13	−125.29 (13)	C29—C28—C33—C38	−133.28 (12)
C4—C5—C6—C1	−0.56 (18)	C29—C30—C31—C26	0.09 (18)
C4—C5—C20—C21	−121.67 (13)	C29—C30—C45—C46	−122.39 (13)
C4—C5—C20—C25	59.68 (17)	C29—C30—C45—C50	58.50 (17)
C4—C14—C15—C16	177.87 (12)	C29—C39—C40—C41	179.43 (12)
C4—C14—C19—C18	−178.55 (12)	C29—C39—C44—C43	179.70 (12)
C5—C4—C14—C15	64.94 (16)	C30—C29—C39—C40	64.06 (16)
C5—C4—C14—C19	−116.33 (13)	C30—C29—C39—C44	−115.95 (13)
C5—C20—C21—C22	−179.26 (12)	C30—C45—C46—C47	−179.88 (11)
C5—C20—C25—C24	177.97 (12)	C30—C45—C50—C49	179.26 (12)
C6—C1—C2—C3	−0.76 (18)	C31—C26—C27—C28	−2.24 (18)
C6—C5—C20—C21	59.24 (16)	C31—C30—C45—C46	58.67 (16)
C6—C5—C20—C25	−119.42 (14)	C31—C30—C45—C50	−120.43 (14)
C7—C1—C2—C3	178.93 (12)	C32—C26—C27—C28	176.59 (12)
C7—C1—C6—C5	−178.26 (12)	C32—C26—C31—C30	−176.86 (12)
C8—C3—C4—C5	−176.44 (11)	C33—C28—C29—C30	−176.65 (11)
C8—C3—C4—C14	4.22 (17)	C33—C28—C29—C39	5.22 (17)
C8—C9—C10—C11	0.98 (19)	C33—C34—C35—C36	1.27 (19)
C9—C8—C13—C12	−0.32 (18)	C34—C33—C38—C37	1.50 (18)
C9—C10—C11—C12	−0.13 (19)	C34—C35—C36—C37	0.49 (19)
C10—C11—C12—C13	−0.93 (19)	C35—C36—C37—C38	−1.23 (19)
C11—C12—C13—C8	1.17 (18)	C36—C37—C38—C33	0.22 (19)
C13—C8—C9—C10	−0.74 (18)	C38—C33—C34—C35	−2.24 (18)
C14—C4—C5—C6	178.33 (11)	C39—C29—C30—C31	176.23 (11)
C14—C4—C5—C20	−0.76 (18)	C39—C29—C30—C45	−2.69 (17)
C14—C15—C16—C17	0.8 (2)	C39—C40—C41—C42	1.3 (2)
C15—C14—C19—C18	0.21 (19)	C40—C39—C44—C43	−0.31 (19)
C15—C16—C17—C18	−0.1 (2)	C40—C41—C42—C43	−1.2 (2)
C16—C17—C18—C19	−0.6 (2)	C41—C42—C43—C44	0.4 (2)
C17—C18—C19—C14	0.5 (2)	C42—C43—C44—C39	0.4 (2)
C19—C14—C15—C16	−0.88 (19)	C44—C39—C40—C41	−0.56 (19)
C20—C5—C6—C1	178.56 (11)	C45—C30—C31—C26	179.04 (11)
C20—C21—C22—C23	1.2 (2)	C45—C46—C47—C48	0.72 (19)
C21—C20—C25—C24	−0.70 (19)	C46—C45—C50—C49	0.1 (2)
C21—C22—C23—C24	−0.5 (2)	C46—C47—C48—C49	−0.06 (19)
C22—C23—C24—C25	−0.8 (2)	C47—C48—C49—C50	−0.5 (2)
C23—C24—C25—C20	1.4 (2)	C48—C49—C50—C45	0.5 (2)
C25—C20—C21—C22	−0.58 (19)	C50—C45—C46—C47	−0.75 (18)

### 5-(Bromomethyl)-1,2,3-triphenylbenzene (2) Crystal data

**Table:**

C_25_H_19_Br	*Z* = 2
*M**_r_* = 399.31	*F*(000) = 408
Triclinic, *P*1	*D*_x_ = 1.363 Mg m^−^^3^
*a* = 10.0859 (6) Å	Mo *K*α radiation, λ = 0.71073 Å
*b* = 10.2444 (7) Å	Cell parameters from 22401 reflections
*c* = 11.3564 (7) Å	θ = 2.8–28.4°
α = 65.309 (5)°	µ = 2.12 mm^−^^1^
β = 76.095 (5)°	*T* = 193 K
γ = 66.367 (5)°	Block, colorless
*V* = 972.95 (12) Å^3^	0.50 × 0.40 × 0.25 mm

### 5-(Bromomethyl)-1,2,3-triphenylbenzene (2) Data collection

**Table:**

Stoe IPDS 2T diffractometer	4702 independent reflections
Radiation source: sealed X-ray tube, 12 x 0.4 mm long-fine focus	4072 reflections with *I* > 2σ(*I*)
Plane graphite monochromator	*R*_int_ = 0.033
Detector resolution: 6.67 pixels mm^-1^	θ_max_ = 28.0°, θ_min_ = 2.8°
rotation method scans	*h* = −13→13
Absorption correction: integration [X-Shape (Stoe & Cie, 2021) and X-Red32 (Stoe & Cie, 2023)]	*k* = −13→13
*T*_min_ = 0.617, *T*_max_ = 0.813	*l* = −14→14
22755 measured reflections	

### 5-(Bromomethyl)-1,2,3-triphenylbenzene (2) Refinement

**Table:**

Refinement on *F*^2^	Primary atom site location: structure-invariant direct methods
Least-squares matrix: full	Hydrogen site location: inferred from neighbouring sites
*R*[*F*^2^ > 2σ(*F*^2^)] = 0.028	H-atom parameters constrained
*wR*(*F*^2^) = 0.069	*w* = 1/[σ^2^(*F*_o_^2^) + (0.0285*P*)^2^ + 0.4117*P*] where *P* = (*F*_o_^2^ + 2*F*_c_^2^)/3
*S* = 1.06	(Δ/σ)_max_ = 0.001
4702 reflections	Δρ_max_ = 0.41 e Å^−^^3^
290 parameters	Δρ_min_ = −0.38 e Å^−^^3^
84 restraints	

### 5-(Bromomethyl)-1,2,3-triphenylbenzene (2) Special details

**Table:**

Geometry. All esds (except the esd in the dihedral angle between two l.s. planes) are estimated using the full covariance matrix. The cell esds are taken into account individually in the estimation of esds in distances, angles and torsion angles; correlations between esds in cell parameters are only used when they are defined by crystal symmetry. An approximate (isotropic) treatment of cell esds is used for estimating esds involving l.s. planes.

### 5-(Bromomethyl)-1,2,3-triphenylbenzene (2) Fractional atomic coordinates and isotropic or equivalent isotropic displacement parameters (Å^2^)

**Table:**

	*x*	*y*	*z*	*U*_iso_*/*U*_eq_	Occ. (<1)
Br1	0.95434 (2)	0.68998 (3)	0.09304 (2)	0.04730 (7)	
C1	0.68276 (16)	0.67536 (18)	0.07518 (14)	0.0279 (3)	
C3	0.45011 (15)	0.71019 (17)	0.20467 (14)	0.0249 (3)	
C4	0.46676 (15)	0.55617 (17)	0.23675 (13)	0.0252 (3)	
C5	0.59163 (16)	0.46289 (17)	0.18471 (14)	0.0265 (3)	
C6	0.69677 (16)	0.52465 (18)	0.10428 (14)	0.0292 (3)	
H6	0.7800	0.4621	0.0685	0.035*	
C7	0.79841 (17)	0.7371 (2)	−0.01017 (16)	0.0339 (3)	
H7A	0.7556	0.8491	−0.0538	0.041*	
H7B	0.8395	0.6914	−0.0782	0.041*	
C8	0.31466 (15)	0.81793 (17)	0.24693 (14)	0.0259 (3)	
C9	0.32032 (19)	0.89833 (19)	0.31745 (16)	0.0343 (3)	
H9	0.4112	0.8843	0.3400	0.041*	
C10	0.1933 (2)	0.9994 (2)	0.35506 (18)	0.0452 (4)	
H10	0.1978	1.0522	0.4049	0.054*	
C11	0.0614 (2)	1.0234 (2)	0.32064 (19)	0.0460 (5)	
H11	−0.0248	1.0930	0.3462	0.055*	
C12	0.05446 (18)	0.9462 (2)	0.24889 (19)	0.0415 (4)	
H12	−0.0364	0.9636	0.2241	0.050*	
C13	0.18000 (17)	0.84313 (19)	0.21296 (16)	0.0330 (3)	
H13	0.1743	0.7892	0.1647	0.040*	
C14A	0.3550 (17)	0.490 (2)	0.3181 (13)	0.026 (2)	0.50 (3)
C15A	0.3200 (16)	0.4786 (18)	0.4481 (12)	0.032 (2)	0.50 (3)
H15A	0.3682	0.5161	0.4824	0.039*	0.50 (3)
C16A	0.2167 (14)	0.4134 (14)	0.5300 (11)	0.0344 (19)	0.50 (3)
H16A	0.1947	0.4073	0.6180	0.041*	0.50 (3)
C17A	0.1483 (10)	0.3588 (11)	0.4793 (15)	0.0339 (18)	0.50 (3)
H17A	0.0778	0.3143	0.5327	0.041*	0.50 (3)
C18A	0.1808 (12)	0.3678 (13)	0.3526 (14)	0.0323 (17)	0.50 (3)
H18A	0.1329	0.3290	0.3193	0.039*	0.50 (3)
C19A	0.2827 (14)	0.4327 (17)	0.2722 (12)	0.0251 (16)	0.50 (3)
H19A	0.3036	0.4378	0.1846	0.030*	0.50 (3)
C14B	0.3540 (17)	0.493 (2)	0.3343 (13)	0.025 (2)	0.50 (3)
C15B	0.3300 (14)	0.4972 (16)	0.4580 (10)	0.0238 (16)	0.50 (3)
H15B	0.3793	0.5440	0.4810	0.029*	0.50 (3)
C16B	0.2326 (12)	0.4322 (16)	0.5479 (11)	0.0332 (17)	0.50 (3)
H16B	0.2180	0.4314	0.6340	0.040*	0.50 (3)
C17B	0.1564 (10)	0.3684 (11)	0.5141 (14)	0.0319 (18)	0.50 (3)
H17B	0.0916	0.3224	0.5773	0.038*	0.50 (3)
C18B	0.1745 (12)	0.3718 (13)	0.3891 (15)	0.0329 (19)	0.50 (3)
H18B	0.1182	0.3340	0.3638	0.039*	0.50 (3)
C19B	0.2761 (16)	0.4314 (19)	0.3001 (14)	0.033 (2)	0.50 (3)
H19B	0.2922	0.4298	0.2147	0.040*	0.50 (3)
C20	0.61581 (15)	0.29975 (17)	0.21167 (14)	0.0269 (3)	
C21	0.61479 (17)	0.19301 (19)	0.33827 (15)	0.0332 (3)	
H21	0.5968	0.2244	0.4101	0.040*	
C22	0.63976 (18)	0.04182 (19)	0.36014 (16)	0.0350 (4)	
H22	0.6387	−0.0297	0.4467	0.042*	
C23	0.66621 (17)	−0.00542 (18)	0.25649 (17)	0.0337 (3)	
H23	0.6829	−0.1091	0.2718	0.040*	
C24	0.66829 (19)	0.09861 (19)	0.13063 (17)	0.0360 (4)	
H24	0.6867	0.0664	0.0592	0.043*	
C25	0.64342 (18)	0.25058 (19)	0.10846 (15)	0.0327 (3)	
H25	0.6454	0.3214	0.0217	0.039*	
C2	0.55917 (16)	0.76671 (17)	0.12599 (14)	0.0271 (3)	
H2	0.5487	0.8698	0.1068	0.033*	

### 5-(Bromomethyl)-1,2,3-triphenylbenzene (2) Atomic displacement parameters (Å^2^)

**Table:**

	*U*^11^	*U*^22^	*U*^33^	*U*^12^	*U*^13^	*U*^23^
Br1	0.03013 (9)	0.07091 (15)	0.04878 (12)	−0.02408 (9)	0.00427 (7)	−0.02660 (10)
C1	0.0258 (7)	0.0339 (8)	0.0231 (7)	−0.0129 (6)	−0.0008 (5)	−0.0077 (6)
C3	0.0244 (6)	0.0301 (7)	0.0216 (6)	−0.0103 (6)	−0.0026 (5)	−0.0094 (5)
C4	0.0250 (6)	0.0299 (7)	0.0208 (6)	−0.0118 (6)	−0.0024 (5)	−0.0069 (5)
C5	0.0277 (7)	0.0279 (7)	0.0221 (7)	−0.0093 (6)	−0.0030 (5)	−0.0070 (6)
C6	0.0272 (7)	0.0325 (8)	0.0254 (7)	−0.0099 (6)	0.0013 (5)	−0.0104 (6)
C7	0.0309 (7)	0.0399 (9)	0.0291 (8)	−0.0171 (7)	0.0027 (6)	−0.0090 (7)
C8	0.0262 (7)	0.0262 (7)	0.0235 (7)	−0.0105 (6)	−0.0008 (5)	−0.0066 (5)
C9	0.0364 (8)	0.0382 (9)	0.0307 (8)	−0.0119 (7)	−0.0049 (6)	−0.0144 (7)
C10	0.0529 (11)	0.0437 (10)	0.0388 (10)	−0.0100 (8)	−0.0004 (8)	−0.0228 (8)
C11	0.0373 (9)	0.0413 (10)	0.0425 (10)	−0.0035 (8)	0.0077 (7)	−0.0150 (8)
C12	0.0249 (7)	0.0421 (10)	0.0476 (10)	−0.0098 (7)	−0.0001 (7)	−0.0102 (8)
C13	0.0286 (7)	0.0350 (8)	0.0363 (8)	−0.0131 (6)	−0.0032 (6)	−0.0113 (7)
C14A	0.025 (3)	0.022 (3)	0.022 (3)	−0.001 (2)	−0.003 (2)	−0.006 (2)
C15A	0.033 (3)	0.032 (3)	0.037 (3)	−0.012 (2)	−0.006 (2)	−0.015 (2)
C16A	0.037 (4)	0.030 (3)	0.027 (3)	−0.010 (2)	0.002 (2)	−0.005 (2)
C17A	0.028 (2)	0.028 (2)	0.039 (5)	−0.0102 (15)	0.000 (3)	−0.006 (3)
C18A	0.032 (2)	0.027 (2)	0.035 (4)	−0.0122 (18)	−0.001 (3)	−0.008 (3)
C19A	0.026 (2)	0.026 (2)	0.023 (3)	−0.0123 (18)	−0.003 (2)	−0.004 (2)
C14B	0.022 (3)	0.027 (3)	0.023 (4)	−0.013 (3)	0.002 (2)	−0.004 (2)
C15B	0.025 (2)	0.027 (3)	0.017 (2)	−0.0108 (18)	0.0018 (16)	−0.0062 (18)
C16B	0.028 (2)	0.037 (3)	0.029 (3)	−0.0121 (19)	0.003 (2)	−0.008 (2)
C17B	0.028 (2)	0.028 (3)	0.030 (4)	−0.0101 (17)	0.001 (2)	−0.003 (2)
C18B	0.030 (2)	0.030 (2)	0.038 (5)	−0.0119 (17)	0.001 (3)	−0.012 (3)
C19B	0.036 (3)	0.034 (3)	0.027 (4)	−0.011 (2)	−0.001 (3)	−0.011 (3)
C20	0.0236 (6)	0.0270 (7)	0.0266 (7)	−0.0074 (6)	−0.0023 (5)	−0.0073 (6)
C21	0.0338 (8)	0.0328 (8)	0.0254 (7)	−0.0069 (6)	−0.0018 (6)	−0.0081 (6)
C22	0.0330 (8)	0.0301 (8)	0.0301 (8)	−0.0088 (6)	−0.0030 (6)	−0.0016 (6)
C23	0.0299 (7)	0.0264 (8)	0.0434 (9)	−0.0093 (6)	−0.0085 (6)	−0.0087 (7)
C24	0.0428 (9)	0.0335 (8)	0.0354 (9)	−0.0118 (7)	−0.0081 (7)	−0.0143 (7)
C25	0.0393 (8)	0.0314 (8)	0.0255 (7)	−0.0126 (7)	−0.0049 (6)	−0.0070 (6)
C2	0.0276 (7)	0.0287 (7)	0.0268 (7)	−0.0127 (6)	−0.0032 (5)	−0.0083 (6)

### 5-(Bromomethyl)-1,2,3-triphenylbenzene (2) Geometric parameters (Å, º)

**Table:**

Br1—C7	1.9711 (17)	C16A—C17A	1.376 (7)
C1—C2	1.390 (2)	C16A—H16A	0.9500
C1—C6	1.391 (2)	C17A—C18A	1.368 (7)
C1—C7	1.495 (2)	C17A—H17A	0.9500
C3—C2	1.396 (2)	C18A—C19A	1.386 (8)
C3—C4	1.410 (2)	C18A—H18A	0.9500
C3—C8	1.494 (2)	C19A—H19A	0.9500
C4—C5	1.413 (2)	C14B—C15B	1.383 (9)
C4—C14A	1.472 (12)	C14B—C19B	1.384 (9)
C4—C14B	1.525 (11)	C15B—C16B	1.388 (8)
C5—C6	1.396 (2)	C15B—H15B	0.9500
C5—C20	1.493 (2)	C16B—C17B	1.386 (7)
C6—H6	0.9500	C16B—H16B	0.9500
C7—H7A	0.9900	C17B—C18B	1.375 (7)
C7—H7B	0.9900	C17B—H17B	0.9500
C8—C9	1.391 (2)	C18B—C19B	1.390 (9)
C8—C13	1.396 (2)	C18B—H18B	0.9500
C9—C10	1.392 (2)	C19B—H19B	0.9500
C9—H9	0.9500	C20—C25	1.389 (2)
C10—C11	1.375 (3)	C20—C21	1.397 (2)
C10—H10	0.9500	C21—C22	1.386 (2)
C11—C12	1.380 (3)	C21—H21	0.9500
C11—H11	0.9500	C22—C23	1.383 (2)
C12—C13	1.387 (2)	C22—H22	0.9500
C12—H12	0.9500	C23—C24	1.382 (2)
C13—H13	0.9500	C23—H23	0.9500
C14A—C19A	1.393 (9)	C24—C25	1.393 (2)
C14A—C15A	1.397 (9)	C24—H24	0.9500
C15A—C16A	1.403 (9)	C25—H25	0.9500
C15A—H15A	0.9500	C2—H2	0.9500
			
C2—C1—C6	118.69 (13)	C15A—C16A—H16A	121.0
C2—C1—C7	121.13 (14)	C18A—C17A—C16A	120.7 (7)
C6—C1—C7	120.18 (14)	C18A—C17A—H17A	119.7
C2—C3—C4	119.65 (13)	C16A—C17A—H17A	119.7
C2—C3—C8	118.59 (13)	C17A—C18A—C19A	120.9 (7)
C4—C3—C8	121.68 (13)	C17A—C18A—H18A	119.5
C3—C4—C5	119.25 (13)	C19A—C18A—H18A	119.5
C3—C4—C14A	122.2 (7)	C18A—C19A—C14A	120.9 (8)
C5—C4—C14A	118.5 (7)	C18A—C19A—H19A	119.5
C3—C4—C14B	118.5 (7)	C14A—C19A—H19A	119.5
C5—C4—C14B	122.1 (7)	C15B—C14B—C19B	119.9 (8)
C6—C5—C4	119.29 (14)	C15B—C14B—C4	119.5 (8)
C6—C5—C20	118.18 (13)	C19B—C14B—C4	120.6 (9)
C4—C5—C20	122.53 (13)	C14B—C15B—C16B	118.9 (8)
C1—C6—C5	121.69 (14)	C14B—C15B—H15B	120.6
C1—C6—H6	119.2	C16B—C15B—H15B	120.6
C5—C6—H6	119.2	C17B—C16B—C15B	121.0 (7)
C1—C7—Br1	110.53 (11)	C17B—C16B—H16B	119.5
C1—C7—H7A	109.5	C15B—C16B—H16B	119.5
Br1—C7—H7A	109.5	C18B—C17B—C16B	119.9 (7)
C1—C7—H7B	109.5	C18B—C17B—H17B	120.0
Br1—C7—H7B	109.5	C16B—C17B—H17B	120.0
H7A—C7—H7B	108.1	C17B—C18B—C19B	119.2 (7)
C9—C8—C13	118.62 (14)	C17B—C18B—H18B	120.4
C9—C8—C3	120.92 (13)	C19B—C18B—H18B	120.4
C13—C8—C3	120.44 (14)	C14B—C19B—C18B	120.9 (8)
C8—C9—C10	120.22 (16)	C14B—C19B—H19B	119.6
C8—C9—H9	119.9	C18B—C19B—H19B	119.6
C10—C9—H9	119.9	C25—C20—C21	118.48 (14)
C11—C10—C9	120.53 (18)	C25—C20—C5	119.49 (13)
C11—C10—H10	119.7	C21—C20—C5	122.01 (14)
C9—C10—H10	119.7	C22—C21—C20	120.65 (15)
C10—C11—C12	119.86 (16)	C22—C21—H21	119.7
C10—C11—H11	120.1	C20—C21—H21	119.7
C12—C11—H11	120.1	C23—C22—C21	120.27 (15)
C11—C12—C13	120.09 (17)	C23—C22—H22	119.9
C11—C12—H12	120.0	C21—C22—H22	119.9
C13—C12—H12	120.0	C24—C23—C22	119.79 (15)
C12—C13—C8	120.66 (16)	C24—C23—H23	120.1
C12—C13—H13	119.7	C22—C23—H23	120.1
C8—C13—H13	119.7	C23—C24—C25	120.02 (15)
C19A—C14A—C15A	116.7 (9)	C23—C24—H24	120.0
C19A—C14A—C4	122.4 (9)	C25—C24—H24	120.0
C15A—C14A—C4	120.9 (9)	C20—C25—C24	120.78 (15)
C14A—C15A—C16A	122.8 (8)	C20—C25—H25	119.6
C14A—C15A—H15A	118.6	C24—C25—H25	119.6
C16A—C15A—H15A	118.6	C1—C2—C3	121.39 (14)
C17A—C16A—C15A	118.0 (7)	C1—C2—H2	119.3
C17A—C16A—H16A	121.0	C3—C2—H2	119.3
			
C2—C3—C4—C5	−2.3 (2)	C4—C14A—C15A—C16A	−178.4 (13)
C8—C3—C4—C5	174.47 (13)	C14A—C15A—C16A—C17A	0 (2)
C2—C3—C4—C14A	−179.4 (7)	C15A—C16A—C17A—C18A	0.1 (15)
C8—C3—C4—C14A	−2.7 (7)	C16A—C17A—C18A—C19A	−0.3 (16)
C2—C3—C4—C14B	173.6 (7)	C17A—C18A—C19A—C14A	0 (2)
C8—C3—C4—C14B	−9.7 (7)	C15A—C14A—C19A—C18A	0 (2)
C3—C4—C5—C6	1.0 (2)	C4—C14A—C19A—C18A	178.2 (13)
C14A—C4—C5—C6	178.2 (7)	C3—C4—C14B—C15B	−58.3 (17)
C14B—C4—C5—C6	−174.7 (7)	C5—C4—C14B—C15B	117.4 (13)
C3—C4—C5—C20	−178.68 (13)	C3—C4—C14B—C19B	121.8 (14)
C14A—C4—C5—C20	−1.5 (7)	C5—C4—C14B—C19B	−62.5 (18)
C14B—C4—C5—C20	5.7 (7)	C19B—C14B—C15B—C16B	3 (2)
C2—C1—C6—C5	−1.2 (2)	C4—C14B—C15B—C16B	−176.7 (13)
C7—C1—C6—C5	179.33 (14)	C14B—C15B—C16B—C17B	−2 (2)
C4—C5—C6—C1	0.8 (2)	C15B—C16B—C17B—C18B	−1.3 (16)
C20—C5—C6—C1	−179.55 (14)	C16B—C17B—C18B—C19B	3.9 (16)
C2—C1—C7—Br1	95.19 (15)	C15B—C14B—C19B—C18B	−1 (3)
C6—C1—C7—Br1	−85.38 (16)	C4—C14B—C19B—C18B	179.2 (13)
C2—C3—C8—C9	−58.5 (2)	C17B—C18B—C19B—C14B	−3 (2)
C4—C3—C8—C9	124.71 (16)	C6—C5—C20—C25	−53.9 (2)
C2—C3—C8—C13	119.61 (16)	C4—C5—C20—C25	125.77 (16)
C4—C3—C8—C13	−57.2 (2)	C6—C5—C20—C21	124.68 (16)
C13—C8—C9—C10	1.2 (2)	C4—C5—C20—C21	−55.7 (2)
C3—C8—C9—C10	179.39 (15)	C25—C20—C21—C22	−0.5 (2)
C8—C9—C10—C11	−1.4 (3)	C5—C20—C21—C22	−179.03 (14)
C9—C10—C11—C12	0.4 (3)	C20—C21—C22—C23	0.1 (2)
C10—C11—C12—C13	0.8 (3)	C21—C22—C23—C24	0.3 (2)
C11—C12—C13—C8	−1.0 (3)	C22—C23—C24—C25	−0.2 (2)
C9—C8—C13—C12	0.0 (2)	C21—C20—C25—C24	0.5 (2)
C3—C8—C13—C12	−178.22 (15)	C5—C20—C25—C24	179.16 (15)
C3—C4—C14A—C19A	117.6 (14)	C23—C24—C25—C20	−0.2 (3)
C5—C4—C14A—C19A	−59.5 (17)	C6—C1—C2—C3	−0.1 (2)
C3—C4—C14A—C15A	−64.6 (17)	C7—C1—C2—C3	179.34 (14)
C5—C4—C14A—C15A	118.3 (13)	C4—C3—C2—C1	1.8 (2)
C19A—C14A—C15A—C16A	−1 (2)	C8—C3—C2—C1	−174.98 (13)

## References

[bb1] Agrawal, P. K., Agrawal, C. & Blunden, G. (2021). *Nat. Prod. Commun.* **16**, 1–12.

[bb2] Bondi, A. (1964). *J. Phys. Chem.* **68**, 441–451.

[bb3] Cook, S. D. (2019). *Plant Cell Physiol.* **60**, 243–254. 10.1093/pcp/pcz004 30649529

[bb4] Dolomanov, O. V., Bourhis, L. J., Gildea, R. J., Howard, J. A. K. & Puschmann, H. (2009). *J. Appl. Cryst.* **42**, 339–341.

[bb5] Farrugia, L. J. (2012). *J. Appl. Cryst.* **45**, 849–854.

[bb6] Gagnon, E., Maris, T., Arseneault, P.-M., Maly, K. E. & Wuest, J. D. (2010). *Cryst. Growth Des.* **10**, 648–657.

[bb7] Gao, K., Xu, A., Krul, C., Venema, K., Liu, Y., Niu, Y., Lu, J., Bensoussan, L., Seeram, N. P., Heber, D. & Henning, S. M. (2006). *J. Nutr.* **136**, 52–57. 10.1093/jn/136.1.52 16365058

[bb8] Groom, C. R., Bruno, I. J., Lightfoot, M. P. & Ward, S. C. (2016). *Acta Cryst.* B**72**, 171–179. 10.1107/S2052520616003954 PMC4822653 27048719

[bb9] Hofer, L. J. E. & Peebles, W. C. (1951). *Anal. Chem.* **23**, 690–695.

[bb10] Jiao, M., He, W., Ouyang, Z., Shi, Q. & Wen, Y. (2022). *Front. Microbiol.* **13**, 964019–964036. 10.3389/fmicb.2022.964019 PMC9493321 36160191

[bb11] Mazik, M. (2022). *ChemMedChem* **17**, e202200157. 10.1002/cmdc.202200157 PMC9321678 35489042

[bb12] Mazik, M. & Seidel, P. (2024). *Molbank* **2024**, M1837.

[bb13] Olthof, M. R., Hollman, P. C. H., Buijsman, M. N. C. P., van Amelsvoort, J. M. M. & Katan, M. B. (2003). *J. Nutr.* **133**, 1806–1814. 10.1093/jn/133.6.1806 12771321

[bb14] Perez, V. C., Zhao, H., Lin, M. & Kim, J. (2023). *Plants* **12**, 266. 10.3390/plants12020266 PMC9867223 36678978

[bb15] Seidel, P., Gottwald, F., Meier, E. & Mazik, M. (2024). *Acta Cryst.* E**80**, 1198–1201. 10.1107/S2056989024009976 PMC11660473 39712148

[bb16] Sheldrick, G. M. (2008). *Acta Cryst.* A**64**, 112–122. 10.1107/S0108767307043930 18156677

[bb17] Sheldrick, G. M. (2015*a*). *Acta Cryst.* A**71**, 3–8.

[bb18] Sheldrick, G. M. (2015*b*). *Acta Cryst.* C**71**, 3–8.

[bb19] Stoe & Cie (2021). *X-SHAPE*. Stoe & Cie, Darmstadt, Germany.

[bb20] Stoe & Cie (2023). *X-RED32*. Stoe & Cie, Darmstadt, Germany.

[bb21] Stoe & Cie (2024*a*). *LANA*. Stoe & Cie, Darmstadt, Germany.

[bb22] Stoe & Cie (2024*b*). *X-AREA*. Stoe & Cie, Darmstadt, Germany.

[bb23] Vardanyan, R. S. & Hruby, V. J. (2006). *Synthesis of Essential Drugs* pp. 19–55. Amsterdam: Elsevier.

[bb24] Westrip, S. P. (2010). *J. Appl. Cryst.* **43**, 920–925.

